# Physical models and simulators in veterinary education: current status, learning impact, and future perspectives

**DOI:** 10.3389/fvets.2026.1774849

**Published:** 2026-04-17

**Authors:** Anna Suñol, Dafni Sivolapenko, Aitor Fernandez-Novo, Megan Madden

**Affiliations:** 1 Department of Veterinary Medicine, Faculty of Biomedical and Health Sciences, Universidad Europea de Madrid, Madrid, Spain; 2 Hospital Veterinaria del Mar, IVC Evidensia, Barcelona, Spain; 3 Department of Neurology, AlphavetWork, Athens, Greece; 4 Hospital for Small Animals, Royal (Dick) School of Veterinary Studies, University of Edinburgh, Edinburgh, United Kingdom

**Keywords:** 3D printing, augmented reality, mannequins, teaching, virtual reality

## Abstract

Veterinary education is undergoing a significant transformation, driven by the increasing integration of physical models and simulation-based training into academic curricula. This review highlights the evolving role of these tools in veterinary programs, encompassing both undergraduate and postgraduate levels. The authors describe and categorize the differences between models and simulators and explore their global and temporal adoption. It describes the application across disciplines and their ability to replicate clinical scenarios, surgical procedures, and diagnostic techniques, also differentiating between students targeted (veterinary medicine training vs. veterinary surgeons). It then evaluates evidence for their educational effectiveness across veterinary disciplines, including comparative analyses with traditional teaching methods when available. Across multiple domains, model and simulation-based training demonstrates equivalent or superior short-term learning outcomes, particularly for anatomy, procedural skills, selected surgical techniques, and emergency interventions, while also offering benefits such as standardized learning experiences, individual satisfaction and reduced learner anxiety. The authors delve into the key challenges of physical models and simulation-based training as well as current limitations of these tools, and future perspectives in development and use of new models for teaching. Ultimately, simulation represents a paradigm shift in veterinary education, aligning ethical responsibility with academic excellence and clinical preparedness.

## 1 Introduction

Veterinary medicine education has traditionally placed emphasis on experiential learning. Students acquire essential competencies through direct engagement with anatomical specimens, surgical procedures, and clinical interventions (1). Historically, this hands-on approach has depended heavily on the use of cadavers and live animals to cultivate both technical proficiency and foundational anatomical knowledge. Cadaveric material, in particular, has played a pivotal role in anatomical education, offering irreplaceable insights for clinical application (2). However, the continued reliance on cadavers presents a range of ethical, logistical, and pedagogical challenges (3–8). The use of live, client-owned animals presents additional considerations relating to ethics, consent, and animal welfare (9–11). In response to these pressing challenges, veterinary education is evolving with efforts to investigate and implement alternative educational strategies that comply with modern standards and uphold both pedagogical rigor and ethical responsibility (12–15).

From simple anatomical replicas to sophisticated virtual reality (VR) platforms, novel training solutions have gained increased significance in veterinary medicine in the last two decades (14, 16, 17). These alternatives create realistic anatomical representations and procedural scenarios without the ethical and practical concerns tied to animal use (15, 18). Models such as three-dimensional (3D) printed anatomical structures, VR simulations, and interactive mannequins have begun to supplement, and in some cases, replace traditional training methods. While limitations exist, they offer benefits such as reduced stress for students during educational interactions and opportunities for repeated practice in a standardized manner (19). This transition not only preserves animal welfare but also responds to evolving societal and technological expectations in veterinary education. In addition, such alternative teaching methods may be more adaptive to students’ diverse learning needs thus providing more inclusive opportunities for learning of practical skills (6, 15). Their integration into veterinary curricula reflects a broader shift toward competency-based education and humane training practices.

Despite its pedagogical advantages, simulation-based training in medical education encounters several persistent challenges. Financial limitations, the technological advances required to develop realistic models and simulators, lack of the clinical realism necessary to foster meaningful skill development or effective implementation among others, render their integration, accessibility and availability difficult across institutions (20). Moreover, questions remain about the effectiveness of simulation-based methods compared to traditional teaching techniques, as well as how best to measure their impact on learning outcome, highlighting a critical gap in the current evidence base and setting the stage for further investigation (20).

The objective of this review is to critically examine the current role of physical models and simulation-based tools in veterinary education. Specifically, the review aims to:

1. Describe and classify the range of physical models and simulators currently used in veterinary education.
2. Evaluate the available evidence regarding the educational effectiveness of these tools across veterinary disciplines and levels of training, and the learning outcomes in comparison with traditional teaching methods, when available.
3. Identify limitations and gaps in the existing literature, including financial, technical, pedagogical, and evidentiary constraints, as well as the lack of data on long-term competency and clinical outcomes.
4. Discuss future directions for the development and integration of simulation-based training in veterinary curricula, incorporating the authors’ interpretations and expectations for different areas of veterinary practice.

To address these objectives, the review is structured to first outline key concepts and classifications, followed by an analysis of applications across veterinary disciplines, an evaluation of educational impact, a discussion of challenges and research gaps, and a forward-looking perspective on future developments (Table 1).

**Table 1:** Mapping of review objectives to manuscript sections and key outcomes.

Objective	Manuscript sections addressing the objective	Key outcomes and limitations
Objective 1: describe and classify current models and simulators	Current use of physical models and simulators	Clear categorization of models by fidelity, complexity, and behavior; however, overlap between categories and inconsistent terminology across studies remain challenging.
Objective 2: evaluate educational effectiveness and comparison with traditional methods	Applications across disciplines Impact on learning and skill acquisition	Simulation generally demonstrates equal or superior short-term learning outcomes compared with traditional methods for anatomy, basic clinical skills, and selected surgical procedures; direct side-by-side comparisons and standardized outcome measures remain limited.
Objective 3: identify limitations and evidence gaps	Impact on learning and skill acquisition Challenges and limitations	Major constraints include cost, realism, faculty training requirements, and limited access; notably, there is a lack of longitudinal data assessing transferability of skills and long-term clinical competency.
Objective 4: discuss future directions and authors’ perspectives	Future perspectives Conclusion	Anticipated growth in AI-driven simulation, 3D/4D printing, and immersive VR/AR tools; uncertainty remains regarding scalability, global equity, and the extent to which simulation can replace live-animal clinical exposure.

## 2 Physical models and simulators: general concepts and proposed classification

This section addresses Objective 1 of the review. It introduces general concepts and terminology surrounding the use of models and simulators in human and veterinary education, outlines existing classifications and provides a structured overview of the existing tools. The authors’ goal is to clarify the correct terminology and to help with a structured framework to improve searchability and comparison between models.

### 2.1 Model vs. simulator

A *model* is defined as a conceptual, mathematical, or physical representation of a system or process. A *simulator*, on the other hand, is a tool that runs a model dynamically to show how it behaves over time or under different scenarios (21). While sometimes they are used interchangeable and the separation line between both is difficult, they should not be used as synonyms. As a summary, models help students visualize and understand, while simulators let them explore and experiment (21). The terminology for models and simulators including purpose, benefits and examples, is reviewed in Table 2.

**Table 2:** A brief review of the terminology for models and simulators.

Term	Definition	Purpose	Benefits	Example
Model	A conceptual, mathematical, or physical representation of a system or process.	To help students understand the structure, components, and relationships within a system.	Encourages abstract thinking and conceptual understanding. Useful for static analysis and prediction and can be physical (e.g., 3D models) or virtual (e.g., flowcharts).	A diagram of the brain (21).
Simulator	A tool that runs a model dynamically to show how it behaves over time or under different scenarios.	To allow learners to interact with the model, test hypotheses, and observe outcomes. Requires a model to function.	Promotes experiential learning and engagement. Is a dynamic format ideal for exploring cause-and-effect relationships and helps students develop decision-making and problem-solving skills.	A virtual brain that responds to movements and drugs (21).

### 2.2 Classification

Simulation tools in veterinary medicine education can be classified into distinct categories (22, 23). Fidelity is defined as the degree of realism or authenticity (24).

Based on their *fidelity*:

- *Low-fidelity models/simulators* are used to teach specific skills; these include suturing pads, palpation trainers, static anatomical replicas, fine needle aspiration of peripheral lymph nodes, jugular venipuncture, cephalic venipuncture, intravenous catheterization, and cystocentesis (25). They are widely utilized in the veterinary curriculum, clinical laboratories and practical skills training and examination (e.g., Objective Structured Clinical Examination), allowing students to practice fundamental skills in a controlled and repetitive environment without the ethical concerns associated with direct animal involvement (26). These tools provide essential hands-on experience and help bridge the gap in learning opportunities due to limited access to live patients (27, 28).
- *High-fidelity simulators* incorporate dynamic features like physiological responses and interactive feedback, exemplified by robotic animals and immersive VR platforms. Robotic dog manikins that simulate breathing, pulse, heart sounds, and even seizures are used for cardiopulmonary resuscitation (CPR), anesthesia, and emergency care training. Virtual reality surgery platforms allow students to perform procedures in a fully immersive digital environment, with haptic feedback (type of digital feedback showing tactile sensations delivered through technology (e.g., vibrations, resistance, or simulated textures) that mimic real-world touch experiences) and real-time error tracking. Advanced equine simulators replicate horse anatomy and physiological responses for training in equine medicine and surgery. These modern tools enhance the realism of training, simulating real-life scenarios to improve skill acquisition and confidence among students, thereby contributing to more humane educational practices (19, 24, 27, 29).
- *Hybrid models/simulators* combine physical components with digital interfaces, enhancing realism and engagement, such as the canine CPR mannequin with scenario software: a combined basic physical model with a tablet interface that lets instructors change heart rhythms, breathing patterns, or simulate cardiac arrest (30). Hybrid scenarios offer a balance between realism and affordability. These models capitalize on the benefits of both static and dynamic formats, offering a comprehensive approach to skill development (19, 24).

Complexity refers to the materials used to build the model/simulator and the number and intricacy of elements involved in the design (31).

Based on their *complexity*:

- Synthetic models:

- Simple models: made from materials such as silicone, latex, and plastic, allowing for a tactile experience that mirrors real-life situations (15, 25).
- 3D printed models (3Dp): Three-dimensional printing, also known as *additive manufacturing*, is a quick and inexpensive technology that creates physical objects from digital files. It was first developed in 1984, but different improvements made it worldwide available and affordable in the last twenty years (31–33). 3Dp technology has emerged as a valuable tool for creating accurate anatomical models that facilitate learning in veterinary medicine (14, 34, 35).
- Integration of digital technology:

- Virtual reality (VR): refers to an integrated set of technologies designed to immerse users in digitally constructed environments. These experiences go beyond simple interaction, engaging the user’s natural senses—such as sight, sound, and touch—to create a convincing sense of presence. At its core, VR enables the simulation of three-dimensional spaces that users can actively explore and manipulate, offering a dynamic and responsive digital world (17, 36, 37).
- Augmented reality (AR): enhances the physical world by superimposing digital elements—such as images, sounds, or data—onto the user’s real-time environment. This fusion of real and virtual content creates an enriched sensory experience that is interactive, responsive, and anchored in three-dimensional space. AR operates in real time, allowing users to engage with both tangible surroundings and computer-generated enhancements simultaneously (17, 36).
- Mixed reality: combination of physical and digital simulators, allowing users to interact with real-world objects enhanced by virtual components. Unlike VR, which fully immerses users in a digital space, or AR, which overlays digital content onto the real world, mixed reality enables seamless interaction between physical models and digital simulations. This hybrid approach enhances realism and engagement, making it particularly valuable in veterinary and medical education. Mixed reality systems often use headsets, sensors, and spatial mapping to anchor digital content to physical objects, enabling learners to manipulate and explore anatomical structures with both tactile and visual feedback. Example: platforms simulating neurological responses on physical models, helping students learn diagnostic techniques by observing virtual symptoms mapped onto real-world replicas (38–40).

Finally, models can be classified based on their *behavior* (41–43).

- Static models: emphasize structure and design. They focus on the architecture of the model. Example: anatomical mannequin.
- Dynamic models: focus on the behavior, interactions, and evolution of a system over time. Example: high fidelity simulators.

**Figure 1:**
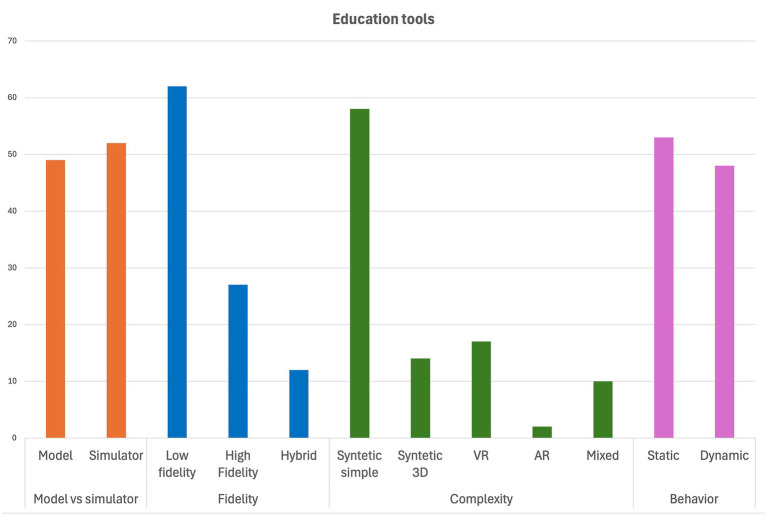
Classifies all the educational tools integrating this review by type (this includes original articles and excludes the reviews). Further information is available in Supplementary Table 1.

Table 3 summarizes the different types of models and simulators, their advantages and disadvantages. Figure 1 classifies all the educational tools (from original articles) integrating this review by types. Further information is available in Supplementary Table 1.

**Table 3:** Summary of the different types of models, their advantages and disadvantages.

Model type	Description	Examples	Applications	Advantages	Limitations	Time to construct	Difficulty of fabrication	Cost estimate (USD)
Low-fidelity models	Static tools for basic procedural skills	Suturing pads, palpation trainers, anatomical replicas	Venipuncture, catheterization, cystocentesis, FNA of lymph nodes	Ethical, cost-effective, easily reproducible	Limited realism and feedback	Hours to days	Low	$ 10–200
High-fidelity simulators	Dynamic models with physiological responses and feedback	Robotic dog manikins, VR surgery platforms, equine simulators	CPR, anesthesia, emergency care, equine surgery	Realistic, immersive, humane	Expensive, technical support needed	Months	High	$ 5,000–100,000 (or more)
Hybrid models	Combines physical models with digital interfaces	CPR mannequins with scenario software	Emergency scenarios, cardiac arrest training	Balanced realism and affordability	Moderate complexity and cost	Weeks to months	Medium to High	$ 500–10,000
Synthetic simple models	Tactile replicas made from silicone, latex, or plastic	Organ replicas, joint models	Suturing, palpation, injection techniques	Durable, tactile feedback	Static, no physiological simulation	Days to weeks	Medium	$ 50–500
3D printed models (3Dp)	Anatomical replicas from digital files using additive manufacturing	Custom organ models, skeletal structures	Anatomy teaching, surgical planning	Customizable, low-cost, rapid prototyping	Limited mobility	Hours to days	Low to Medium	$ 20–300
Virtual reality (VR)	Immersive digital environments with sensory feedback	VR surgery platforms, interactive anatomy labs	Surgery, diagnostics, procedural training	High engagement, error tracking	Requires hardware, high initial cost	Months	High	$ 10,000–150,000 (or more)
Augmented reality (AR)	Digital overlays on real-world environments	AR anatomy apps, interactive procedure guides	Anatomy, diagnostics, procedural walkthroughs	Real-time interaction, enhanced visuals	Less immersive than VR	Weeks to months	Medium to High	$ 5,000–50,000
Mixed reality	Combines physical and digital simulation tools	AR-enhanced manikins, VR with haptic devices	Multi-modal training across disciplines	Combines strengths of all formats	Complex setup, integration challenges	Months	High	$ 20,000–$ 200,000 (or more)

### 2.3 How to select a model or simulator?

Having outlined the range and classification of available simulation tools, this subsection synthesizes the practical considerations for selecting appropriate models and simulators.

**Figure 2:**
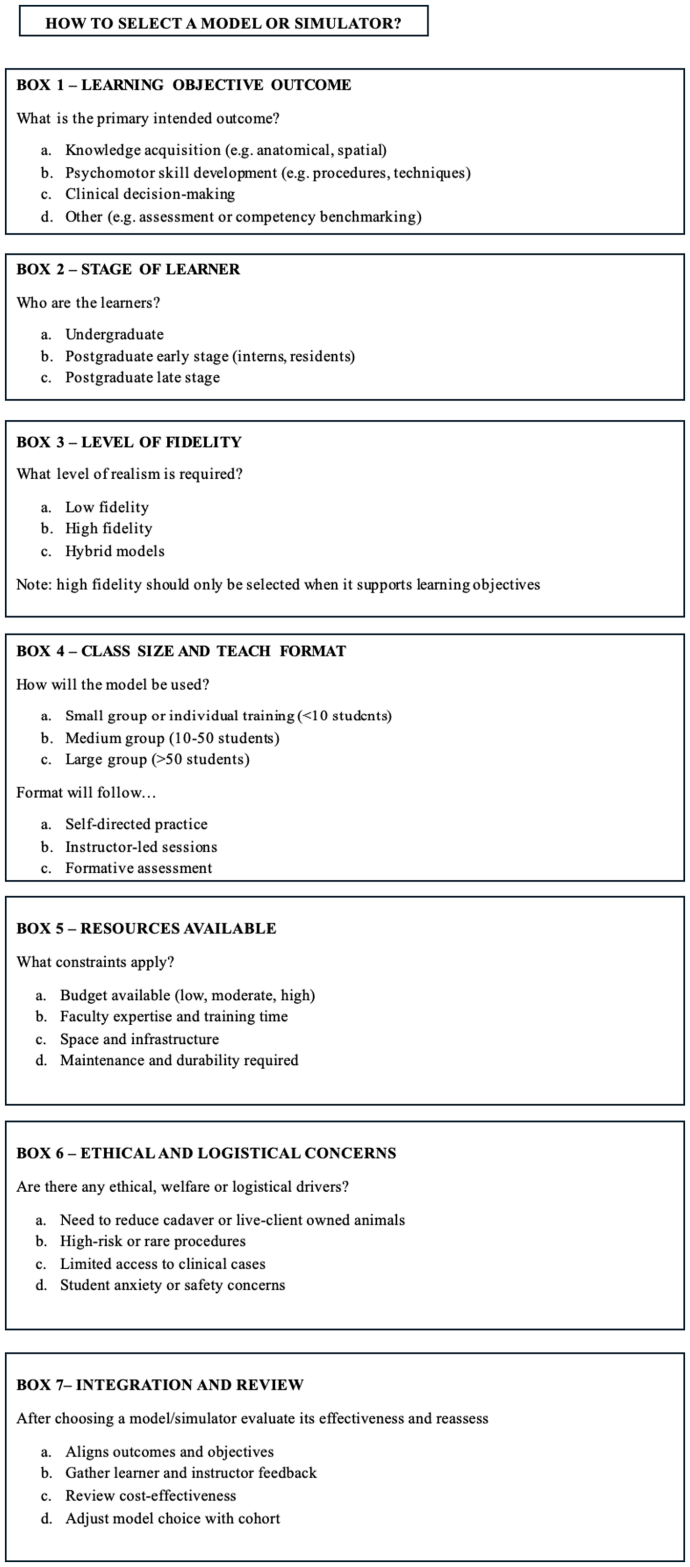
Includes some recommended considerations made by the authors for selecting physical models and simulators in veterinary education based on educational context and learning objectives.

Based on the evidence reviewed and the authors’ experience in veterinary education, the selection of an appropriate model or simulator should be guided by clearly defined learning objectives rather than by fidelity or technological sophistication alone. In the authors’ view, and supported by the reviewed literature, the most educationally effective teaching tools are not necessarily the most expensive or technologically advanced (25, 44, 45). The authors consider that educators should prioritize alignment between the intended learning outcome and the simulator’s functional characteristics, rather than adopting high-fidelity tools by default. For instance, what type of procedure they want to replicate (e.g., whether it relies on anatomical landmarks or dynamic learning), or its complexity and relevance in a clinical scenario (e.g., a simple procedure such as fine needle aspiration samples vs. CPR). Additionally, the teachers will need to consider how they are going to instruct it (e.g., full class of students, small groups or one-to-one) as well as their resources, including not only financial investment but also allocated time for the activities and educators’ experience. For example, if a large number of students are taught a non-complex procedure, a low-cost, durable and easily reproducible, model may be preferred; this can save time compared to students learning with a single machine (example—virtual reality glasses) with one student using them at a time. Figure 2 includes some criteria considered by the authors for selecting physical models and simulators in veterinary education based on educational context and learning objectives. Further research is needed to know which type of model is best for each category to be able to create a diagram flowchart from these considerations in the future.

### 2.4 Global and temporal adoption

**Figure 3:**
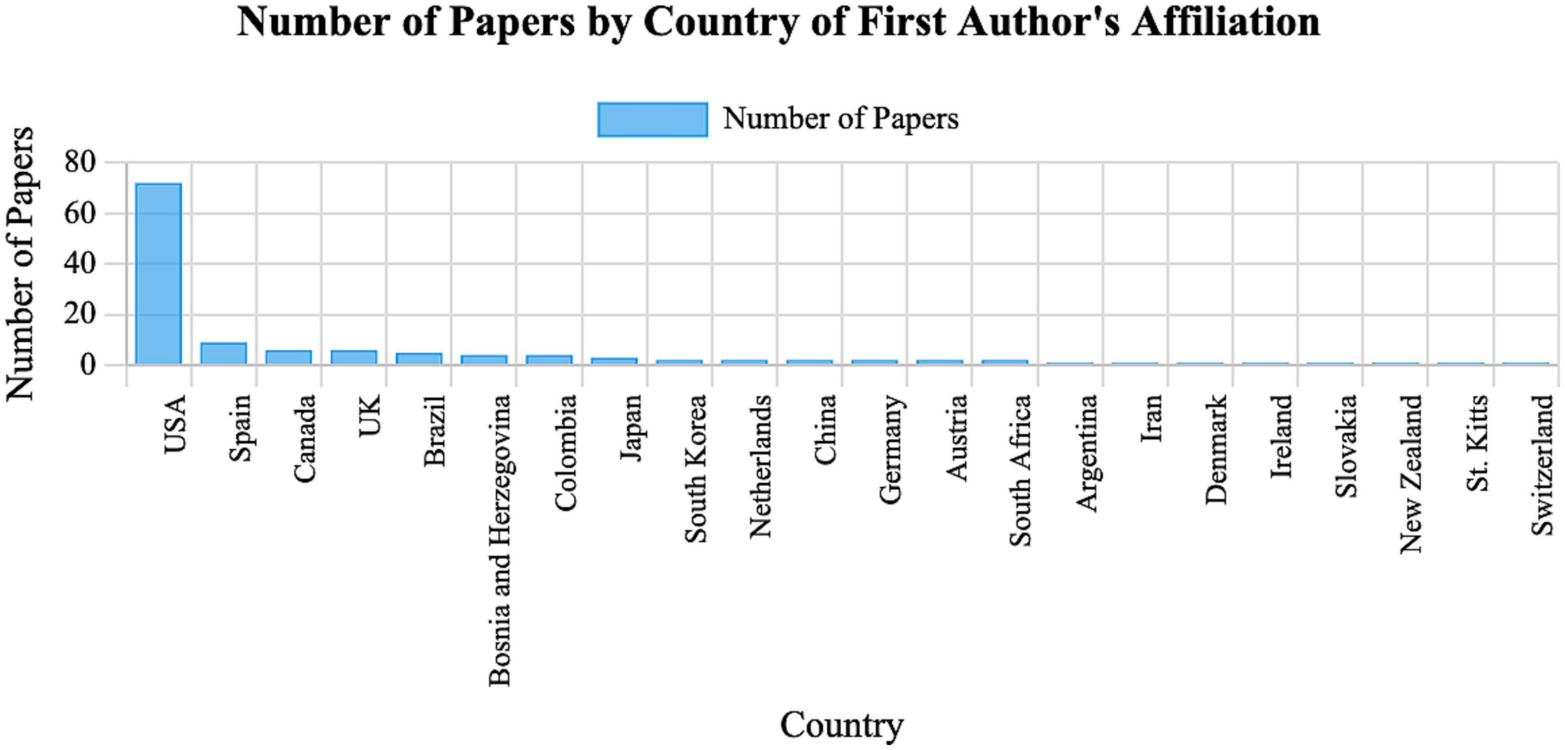
Classification of all the articles integrated in this review by country of the first author (this includes not only the original articles but also the reviews). This information is available in more detail as a table in Supplementary Table 2. The figure and table have been generated using an artificial intelligence tool [Claude AI by Anthropic running Sonnet 4.5 (https://claude.ai/)].

**Figure 4:**
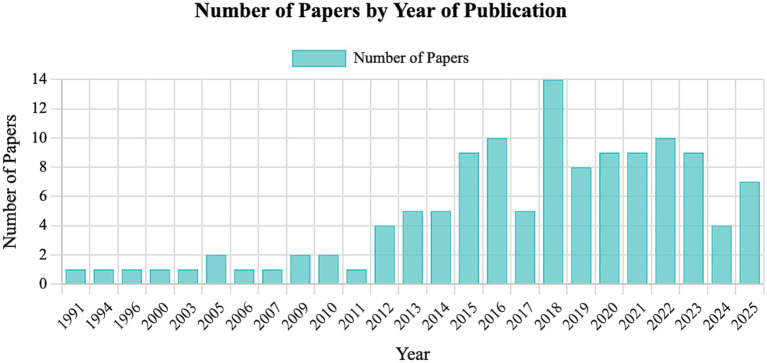
Classification of all articles integrated in this review by year of publication (this includes not only the original articles but also the reviews). This information is available in more detail as a table in Supplementary Table 2. The figure and table have been generated using an artificial intelligence tool [Claude AI by Anthropic running Sonnet 4.5 (https://claude.ai/)].

Several institutions in North America, Europe, and Australia have established simulation centers and integrated models and simulators into curricula (46, 47). However, adoption varies globally due to resource disparities and faculty readiness. Figure 3 classifies all teaching-integrated articles in this review (including reviews) by country of the first author being United States of America the ones with the highest number of publications. Figure 4 classifies all teaching-integrated articles in this review (including reviews)the articles by year of publication (12, 14, 15, 19, 20, 22–28, 34–40, 43–45, 48–151).

The year 2018 was by far the one with more articles in this topic which could be linked to growing accessibility of simulation technologies (e.g., 3Dp, VR) alongside increased emphasis on competency-based education and ethical alternatives to live animal use in veterinary training. This convergence likely stimulated wider adoption and reporting models and simulator-based methodologies during that period. This information is available in more detail as a table in the Supplementary Table 2.

### 2.5 Physical models and simulators: summary points

- Models and simulators should not be used as synonyms. As a summary, models help students visualize and understand, while simulators let them explore and experiment.
- Models and Simulators are similarly used in veterinary education, with low fidelity being most available.
- Based on their complexity, synthetic simple models are by far the most described in the literature.
- Based on their behavior, there is similar availability between static and dynamic tools.
- Educators and future research should prioritize alignment between the intended learning outcome and the simulator’s functional characteristics, rather than adopting low or high-fidelity tools by default.
- United States is the countries with most publications regarding this topic.
- The year with most publications to date have been 2018.

In summary, veterinary simulation tools encompass a diverse and increasingly complex range of models that vary widely in fidelity, complexity, and behavior. While existing classification systems help organize this diversity, overlap between categories and inconsistent terminology across studies complicate direct comparison. Furthermore, lack of model specific description resulted in some articles not being included in this review, as it was not possible to establish if the authors were employing a model or a simulator, or what type at all. In the authors’ view, greater conceptual clarity and more consistent reporting standards are needed to improve interpretability and facilitate meaningful comparison across studies. Establishing a shared framework will be essential as simulation continues to expand within veterinary curricula.

## 3 Applications across disciplines

This section addresses Objective 2 by examining how models and simulators are used across veterinary disciplines and at different levels of training, from undergraduate to postgraduate education. It explores the types of skills taught through simulation-based approaches and evaluates the available evidence for their educational effectiveness within specific clinical and academic contexts. It also aims to identify their limitations and assess how they compare with more traditional instructional methods.

### 3.1 Qualification status of the student

**Figure 5:**
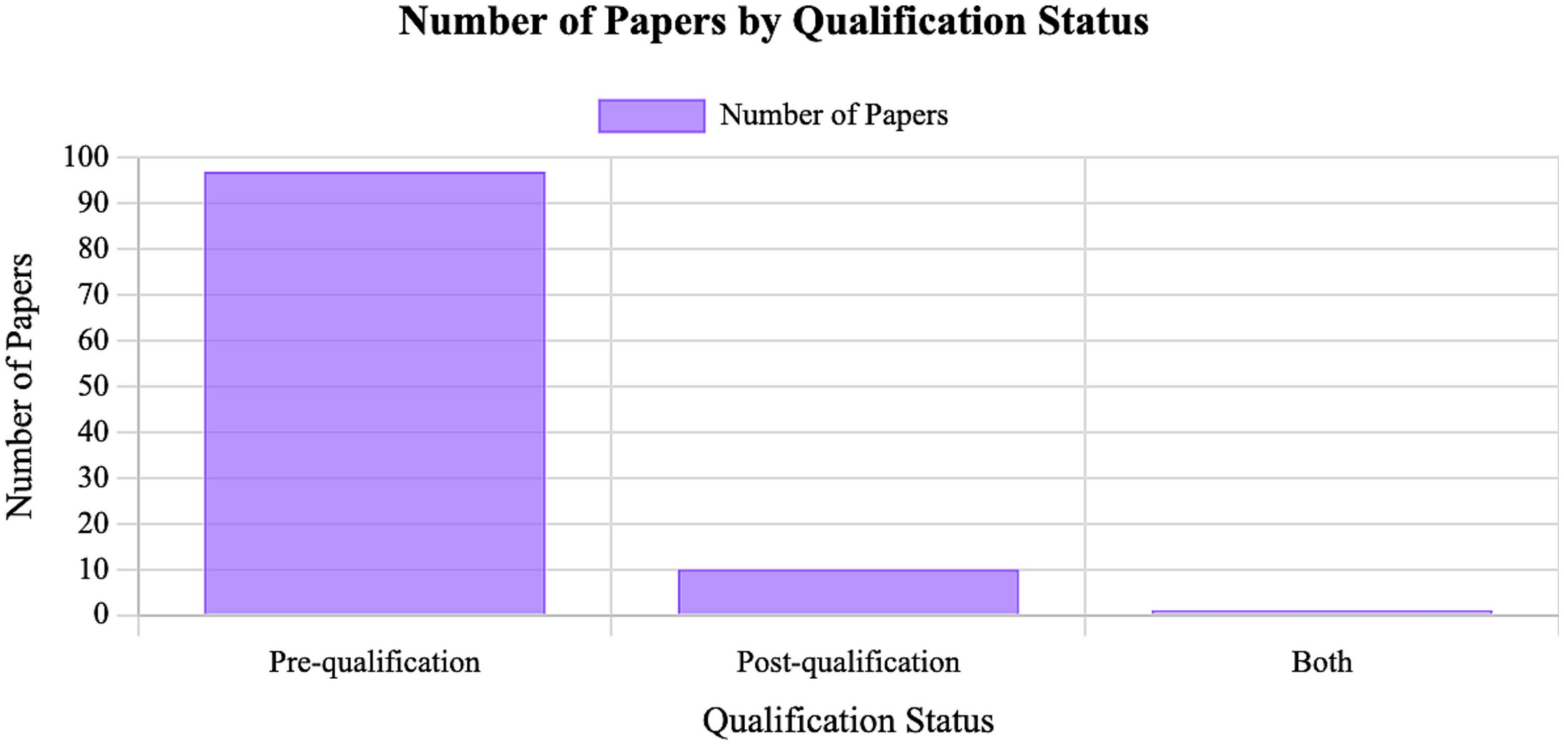
Classification of the articles integrated in this review according to the level of education of the trainee; the “pre-qualification” category includes education during the veterinary degree and the “post-qualification” category includes training for qualified vets (this includes not only the original articles but also the reviews). This information is available in more detail as a table in Supplementary Table 2 [Claude AI by Anthropic running Sonnet 4.5 (https://claude.ai/)].

When considering a simulation-based teaching it is fundamental to decide which type of student will benefit from the learning. Figure 5 classifies the original articles included in this review between qualification status of the student (i.e., whether the teaching took place during veterinary medicine training or after qualification as a veterinary surgeon). Further information is available in Supplementary Table 1 (12, 14, 25, 27, 28, 34, 35, 37, 44, 45, 48–51, 53–71, 73–79, 81, 82, 84–100, 102–125, 127–135, 137–151). Results showed that research in models and simulators for teaching has been primarily focused on undergraduate students. In the author’s opinion this trend is likely influenced by several factors: veterinary medicine training curricula (undergraduate training in most countries) are typically more structured and standardized, the larger student cohorts create greater incentive to optimize teaching methods, and the higher numbers make it easier to conduct studies with adequate sample sizes and generalizable outcomes. These programs also tend to integrate practical skills training earlier and more consistently, increasing the demand for reliable simulation-based tools. Together, these factors most likely contribute to the predominance of undergraduate-focused research and facilitate the extension of findings to veterinary surgeons education.

### 3.2 Species design

**Figure 6:**
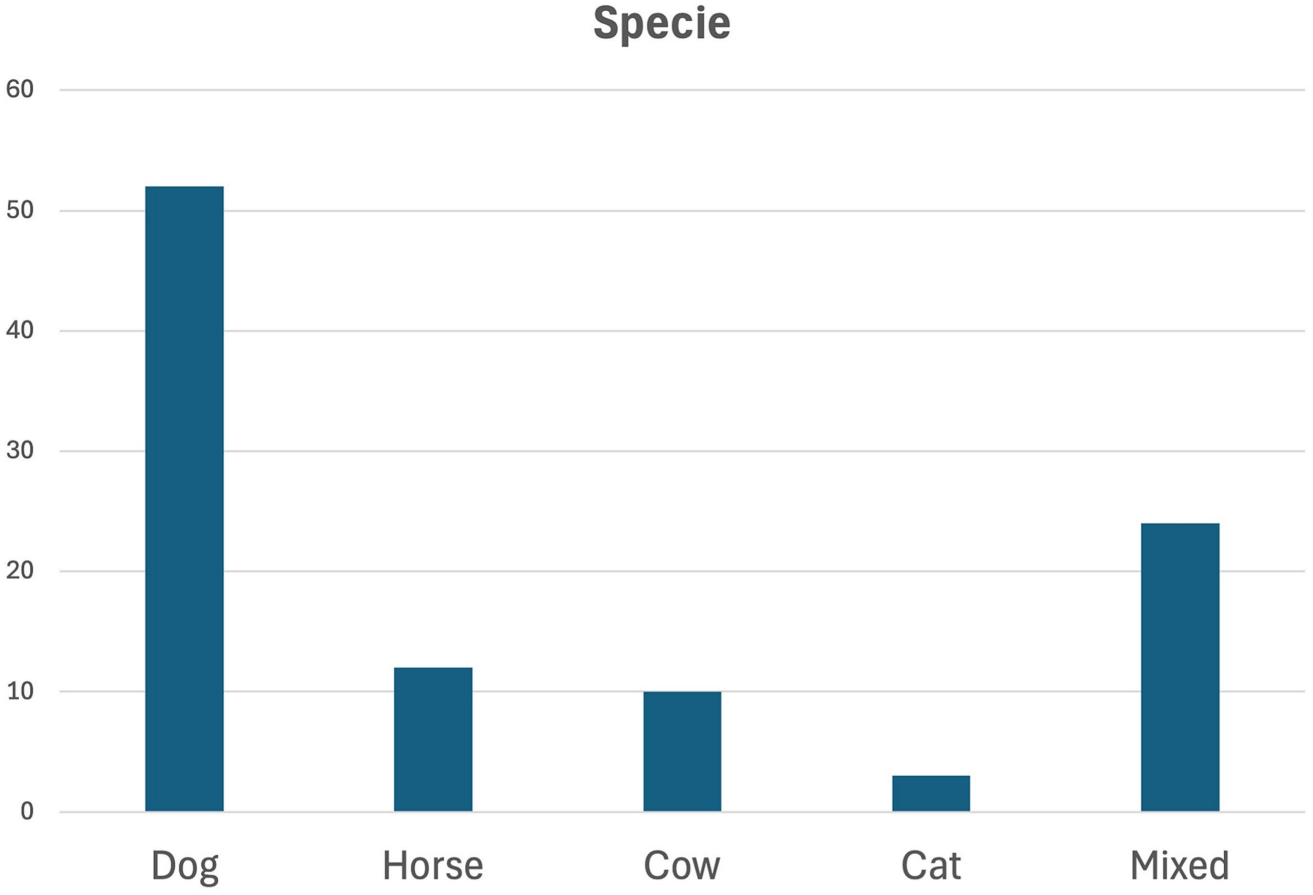
Classification of the articles integrated in this review according to the species the model/simulator waFs targeting. This information is available in more detail as a table in Supplementary Table 1.

Figure 6 classifies the articles included in this review by species targeted. Further information is available in Supplementary Table 1. Results showed that most models and simulators were created for dogs. In the author’s opinion this result is not surprising, as it is the most commonly studied species in veterinary programs.

### 3.3 Anatomy

Traditionally, learning anatomy has relied on cadaveric dissection and the use of textbooks (1). Cadaveric dissection offers hands-on experience and a realistic understanding of anatomy, while textbooks provide structured content and visual illustrations for foundational learning. However, cadaveric dissection can be costly, time-consuming, and ethical and emotionally challenging, while textbooks often lack the dynamic and three-dimensional perspective needed for spatial understanding (1). The limitations and challenges of both techniques created the need for utilization of new ways of teaching (3, 6–8). Commercially available anatomical models, provided one solution to this challenge (39). However, these models are limited in variety. To provide more anatomical variants that are particularly useful for teaching anatomy across different disease contexts in veterinary medicine, the use of 3Dp technology was introduced. 3Dp can replicate more complex anatomical scenarios (e.g., fractures, malformations); or produce specific models that are not commercially available yet. Research has shown that veterinary students benefit from hands-on experiences with 3Dp models, which help improve their understanding of complex anatomical structures. Preece et al. (14) published the first veterinary article using this technology to teach veterinary anatomy using a 3D-printed equine foot. The research found that the use of 3D-printed equine anatomical models led to significant improvements in veterinary students’ assessment scores compared to those relying solely on traditional textbooks or digital models (14). Similarly, other studies indicate that 3Dp models crafted from cadaveric CT scans provide feasible and engaging learning resources, enhancing spatial understanding and conceptual knowledge (109, 136). The study of anatomy using 3Dp is also important in clinical scenarios. In 2019, Suñol et al. (35) evaluated the effectiveness of 3D-printed models for teaching canine spinal anatomy and vertebral fractures. Comparing CT scans, 3D computed reconstructions, and 3Dp models, it was found that students using 3Dp models for this purpose showed superior performance and faster response times than those using CT scans, with no significant time difference between 3Dp and 3D computed reconstruction groups (35). Since then, multiple studies have shown that 3Dp technology can complement traditional anatomy teaching by encouraging spatial understanding. However, it is crucial to note that not all 3Dp models maintain the anatomical detail required for comprehensive learning; some may lack essential details necessary for higher education standards in veterinary anatomy, including vascular, ligamentous or tendinous structures (63, 101, 110, 111, 149, 152).

Furthermore, the application of AR and VR technologies in veterinary anatomy education presents an exciting frontier for interactive learning (17). Augmented reality provides students with a dynamic way to interact with anatomical models in three dimensions, which can be superior to traditional approaches. For instance, Christ et al. demonstrated that AR could enhance students’ understanding of neuroanatomy through its interactive features (68). Furthermore, Jiang et al. (98) reported significant improvements in student comprehension and engagement when AR models of the canine skull were introduced, highlighting the potential for immersive learning experiences that traditional models cannot offer.

Virtual reality, similarly, serves as a powerful instructional tool, allowing students to simulate complex surgical procedures (eg. Mandibular reconstruction) and explore veterinary anatomy in a risk-free environment, allowing learners to reduce surgical time and improve accuracy (153).

Overall, the incorporation of 3D printing, AR, and VR in veterinary anatomy education addresses various challenges faced in traditional teaching methods, such as limited access to cadaveric resources and the need for enhanced engagement in learning. These technologies have demonstrated significant potential to improve educational outcomes, promote better spatial understanding of anatomical structures, and offer innovative solutions to long-standing teaching challenges (17, 31). As research continues to validate their effectiveness compared to traditional techniques, it is clear that these technological advancements are likely to play an increasingly important role in enhancing veterinary education, supporting students as they navigate their future careers in a field that continues to evolve.

In this review from the original papers analyzed 20/101 were focusing on anatomical knowledge. There were minimal differences between types of models/simulators, their fidelity and complexity. However, the majority had a static behavior (14/20). In the authors’ opinion this is an expected finding considering that early stage of training typically prioritizes foundational anatomical understanding over functional pathophysiology.

### 3.4 Clinical examination, organ palpation and non-surgical procedures

Veterinary students are expected to be competent in hundreds of skills, similarly across the globe, to meeting certain standards prior to graduation (e.g., Royal College of Veterinary Surgeons or American Veterinary Medical Association “day one competencies”) (39, 154, 155). Therefore, the use of models and simulators has become a practical way to teach those skills and standardize students’ exposure and performance prior to graduation. Commercial veterinary simulators are available for teaching procedures such as venipuncture, suturing, or endotracheal intubation (39). However, institutions also create their own models and simulators for integration into their clinical skills laboratories.

Other important clinical scenarios include organ palpation. Capilé et al. (64) reported a model for prostatic palpation in dogs. Prostate palpation training allows students to learn how to correctly introduce the finger into the rectum and identify the location, size, symmetry, and consistency of the prostate, without the need of real, and affected, dogs. Similar simulators have been created for bovine rectal palpation of internal reproductive organs, and Baillie et al., also tested a VR haptic model for the same purpose and found that it improved students’ success with identifying the uterus (27, 58, 62). In a study by Mccaw et al. (37) immersive VR was used for students to practice pelvic limb lameness interpretation in dogs.

In 2013, Eichel et al. (73) reported a model to teach techniques for injecting the jugular vein in horses. The training model proved to be a useful tool to teach veterinary students how to perform jugular vein injections in horses in a controlled environment, without time limitations or animal welfare concerns. Similarly, Pérez-Merino et al. (127) compared the usefulness of fresh-frozen canine cadavers and a validated canine simulator model for training veterinary students in basic gastrointestinal endoscopic procedures. The use of the simulator appeared to be a viable alternative to use cadavers for veterinary endoscopic training. However, the cadaver group were faster completing all tasks. This result was considered to be due to the more realistic features of cadavers compared to the simulator, which confused some students. However, the model provided students with a good level of proficiency before performing endoscopic procedures on live dogs (127).

Madden et al. (34) and Langebæk et al. (104) described models to teach cerebrospinal fluid collection in dogs from the cervical and lumbar subarachnoid space, respectively. This technique is typically learnt at a postgraduate level using client-owned pets undergoing diagnostic investigations for neurological disease. Similar models have been used to teach lumbosacral epidural anesthesia techniques (71). Development of these models allows a safe environment for teaching beyond clinical scenarios.

Other simulators include the equine nerve block simulator to supplement practical skills training in the undergraduate veterinary curriculum, a low-cost portable simulator of a domestic cat larynx for teaching endotracheal intubation, a model to teach ultrasound-guided injections of the cervical articular process joints in horses, a model to identify vegetal foreign bodies in canine limbs using ultrasound and models to teach funding examination in dogs, or otoscopic examination among others (60, 69, 90, 121, 122, 142, 153).

Other authors, such as Anderson et al., have developed models for multiple purposes, in an attempt to cover multiple skills with a single model (25). In our opinion such models are promising not only to reduce the need for numerous single-use devices but also optimize limited institutional resources, including budget allocations, physical storage space and improve environmental sustainability with less resources needed. Moreover, multipurpose simulators may foster more holistic learning experiences by allowing students to practice interconnected procedures in a realistic, sequential manner (25).

Furthermore, VR has been used to teach different anesthesia skills, including canine endotracheal intubation technique and assess veterinary students on the use of anesthetic machine. In the first study, the authors developed a VR model and compared their effectiveness with traditional—video tape methods. Results showed similar outcomes than traditional methods and all students enjoyed the experience (150). In the second, VR was found to be a suitable model to teach anesthesia, although it presented some inherent limitations such as cybersickness (102).

In this review from the original papers analyzed 40/101 were created to teach non-surgical clinical skills. There was a similar representation between models and simulators. However, the majority were of low-fidelity (27/40), which was felt appropriate for their teaching purposes. Based on their complexity 23/40 were made with synthetic simple materials and based on their behavior, 23/40 were considered dynamic. These results showed the authors engine to create low cost and easy reproducible tools to teach clinical skills across different disciplines.

### 3.5 Surgery

Surgery, similar to anatomy and clinical skills, is one of the fields where these models are significantly changing the teaching and clinical landscape. The first models were already described in the 90s (133). Simulation laboratories allow repeated practice before training with live animals. In this field there are multiple types of model reported—from low to high fidelity, to models only addressing parts of the animals and specific procedures and models with multiple uses (20, 53, 54, 107, 123).

A wide range of commercially available simulator models are now obtainable and are used to train veterinary surgery, offering realistic and ethical alternatives to live animal practice (20). Low fidelity models such as suturing pads have been used for years (20). In addition, there are several companies that provide high-fidelity models designed for procedures such as suturing, ovariohysterectomy, and castration in several species. These simulators replicate anatomical structures and haptic feedback, enabling students to develop essential surgical skills in a controlled, repeatable environment. Their integration into veterinary curricula supports hands-on learning, reduces student anxiety, and aligns with humane education principles (156).

Moreover, patient-specific 3Dp models have significantly improved pre-surgical planning and education for veterinary trainees, as noted in studies highlighting these models’ ability to enhance comprehension of spatial relationships among structures while reducing the need for cadaver use (157). The engagement levels of students using 3Dp models have also been positively highlighted, showcasing their effectiveness in interactive learning environments (61). In a study focusing on veterinary surgical education, Winer et al. highlight the potential of 3Dp for preoperative planning, asserting that such models not only assist in executing these procedures but also enhance the effectiveness of training through tangible interaction with anatomy (158). As explored by Thomas et al., 3Dp anatomical models permit better pre-surgical evaluations, enabling surgeons to devise tailored surgical plans that improve operating room efficiency and outcome predictability (159). The engagement levels of students using 3D-printed models have also been positively highlighted, showcasing their effectiveness in interactive learning environments (61).

Virtual reality is also being implemented into surgical skill training in veterinary education. Training programs enhanced by virtual and augmented reality are poised to advance surgical skills among veterinarians, providing immersive experiences that replicate the nuances of live procedures without the associated risks (67, 72).

For minimally invasive surgery and laparoscopy, several different types of low and high-fidelity simulators have been developed, including the “Mayo Endoscopy Simulated Images Canine Abdominal model,” the “Canine Laparoscopic Simulator,” the “CALMA Veterinary Lap-Trainer Composite Simulator,” the “Canine Simulated Laparoscopic Ovariectomy Model,” and the “Standing Equine Laparoscopic Ovariectomy Simulator” (66, 72, 74, 79, 80, 94, 124–126, 138, 140). These have been validated enhancing learning outcomes. A study by French et al. (82) found that students who performed well on a high-fidelity laparoscopic simulator for abdominal surgery practicing laparoscopic ovariectomy were able to perform this procedure on a live dog under supervision, suggesting that such simulators can increase readiness for laparoscopic procedures.

In this review, 37/101 original papers were focused on surgical skills. Most tools described were considered to be low fidelity models made of synthetic materials (22/37). These results are similar to what was found for clinical skills teaching, suggesting this type of models is more attractive to researchers and possibly teachers. This could be due to economical or practical reasons most likely.

### 3.6 Diagnostic imaging

In recent years, there has been a growing interest in the use of simulators and advanced technologies for diagnostic imaging in veterinary medicine, both from a teaching perspective to a clinical need. Similar to surgery, this discipline relies on outstanding anatomical knowledge. Hence, the use of 3-dimensional learning either using a screen or 3Dp models can enhance students’ performance (35). Similarly, deep learning frameworks, as discussed by Xiao et al., are not only advancing clinical diagnostics but also providing students with artificial intelligence (AI) tools that improve their understanding of anatomy and complex imaging results (160). Banzato et al. (161) created a technology to detect degenerative disease from ultrasound images. In the study, the authors compared their technology with serum biochemistry and cytology on the same samples, using histopathology as standard. This technique could establish a methodological basis that enhances postgraduate teaching and offers exposure to cutting-edge diagnostic approach to undergraduate students, while improving clinical diagnostics. However, researchers such as Hoscheit et al. (162) underline the importance of quality assurance in B-mode ultrasound imaging, advocating for the establishment of protocols to ensure optimal diagnostic image quality among veterinary ultrasounds.

To the authors’ opinion integration of automated evaluation methods, such as the AI-based algorithms for thoracic radiographs explored by Banzato et al. (163) further illustrates how technological tools could evolve from clinical diagnosis to teaching and incorporated into curricula in the future to improve students’ diagnostic accuracy and confidence. In addition, Gallastegui et al. (84) have demonstrated that implementing case-based learning in veterinary radiology notably improves students’ radiographic interpretation skills, illustrating the effectiveness of active learning strategies. In one study, immersive VR was used to teach orthopedics, including radiographic interpretation, and this was found to improve the students’ clinical reasoning and course information retention (37). Overall, these recent studies demonstrate how the integration of simulators and AI technologies could advance diagnostic imaging training methods in undergraduate and post-graduate veterinary curriculums.

From the articles reviewed, only 3 original articles used students for teaching diagnostic imaging techniques using models or simulators and mainly focused on x-rays and ultrasound skills. In the author’s opinion, this will very likely be one of the areas with more future development in the upcoming years with the use of AI. For the purpose of this review the articles focusing on specific clinical skills using diagnostic imaging (e.g., fine needle aspiration) have been classified in the previous 3.4 Section.

### 3.7 Cardio-pulmonary resuscitation

Innovative learning methodologies are becoming increasingly popular for emergency procedures such as CPR. These procedures demand not only technical precision but also rapid decision-making under intense psychological pressure and conditions that can overwhelm even experienced clinicians. Therefore, structured and immersive training is essential to prepare veterinary students for real-world emergencies as well as provide additional training for already qualified veterinary professionals.

Emergency situations in veterinary practice are inherently stressful, often requiring life-or-death decisions to be made within seconds. Studies have shown that delayed initiation of CPR dramatically reduces survival rates (30). This underscores the critical need for pre-emptive training that builds not only technical competence but also psychological readiness. Training environments that simulate the urgency and unpredictability of real emergencies help students develop resilience and procedural fluency, reducing hesitation and error during actual clinical crises (30). To address this need, anatomically detailed simulation models have been developed to facilitate a deeper understanding of procedural complexities (76, 136). These models are especially valuable for replicating high-pressure situations, similarly to surgical training, allowing students to rehearse critical interventions in a controlled environment (76, 136, 157). Among the most impactful innovations is the integration of RECOVER CPR training, a globally recognized, evidence-based curriculum developed by over 100 board-certified veterinary specialists. The RECOVER Initiative offers structured certification in both Basic Life Support (BLS) and Advanced Life Support (ALS), using standardized algorithms and cognitive aids to teach high-quality CPR techniques for dogs and cats with simulators. These modules include both online and in-person components, ensuring comprehensive skill acquisition. Given that less than 6% of dogs and fewer than 20% of cats survive cardiopulmonary arrest to hospital discharge, the importance of standardized CPR training cannot be overstated (30).

Virtual Reality and AR technologies further revolutionize emergency training. Virtual reality provides a fully immersive platform where trainees can practice CPR and other procedures in lifelike scenarios, enhancing muscle memory and situational awareness without ethical concerns associated with live animal use. Augmented reality complements this by overlaying digital guidance onto physical models, enabling real-time visualization of anatomical landmarks and procedural steps. This hybrid approach bridges the gap between theoretical instruction and hands-on application (136, 164).

Overall, these tools not only enhance technical proficiency but also prepare students to navigate the psychological demands of emergency care, ultimately improving patient outcomes and elevating the standard of veterinary practice.

In the articles included this review only 1 original article used students for teaching CPR using models or simulators. This might be due to the large number of available commercial models for this purpose compared to other disciplines.

### 3.8 Clinical reasoning and communication

Transferable skills are abilities and competencies developed in one context that can be applied effectively across different roles or situations. Examples include communication, teamwork, problem solving and adaptability, among others. Those skills are as crucial as the rest mentioned in this section for veterinary medical education. In one study training veterinary students in orthopedics using VR, client communication scenarios were included (37). Although few articles have explored the use of models and simulators for this purpose, it presents a promising avenue for fostering clinical reasoning among both undergraduate and postgraduate veterinary students (19, 23, 137).

### 3.9 Comparison with traditional teaching methods

Across veterinary education, simulation-based training is most frequently evaluated in comparison with traditional teaching approaches such as didactic instruction, cadaver-based learning, and supervised clinical exposure. However, from all the original articles integrating this review, 36/101 did not compare their model or simulator with any other method. 54/101 compared with traditional methods (the most common being cadavers, videos and books). Finally, 11/101 compared with different models (3Dp, VR, synthetical and commercial). Approximately one third of studies did not compare results to traditional teaching methods, this could be due to different reasons, including ethical concerns, financial constraints and lack of a replicable method. This puts in perspective the results from these models and simulators and highlights their limitations when comparison of their effectivity is made.

Regarding their outcome, in 24/54 of the articles reported superior results with the model or simulator compared to the traditional method, 18/54 reported the same outcome, 8/54 reported variable results and 2/54 described worse outcome compared to traditional methods. In all studies, feedback from participants was very positive and they enjoyed the new teaching tool. Looking into more detail, the reviewed evidence indicates that, for short-term learning outcomes, simulation-based methods generally achieve equivalent or superior performance in knowledge acquisition, procedural accuracy, and learner confidence, particularly for anatomy, basic clinical skills, and selected surgical and emergency procedures. In contrast, traditional methods remain critical for developing contextual clinical reasoning, professional judgement, and adaptability to patient variability, outcomes that are less consistently assessed in simulation studies. In the authors’ view, simulation-based training is best positioned as a preparatory and complementary approach, enabling structured skill acquisition and deliberate practice before clinical exposure, rather than as a replacement for traditional experiential learning. Importantly, while simulation offers advantages in standardization, ethical acceptability, and learner safety, the lack of longitudinal evidence linking simulated performance to long-term clinical competence limits definitive conclusions regarding its comparative superiority. As such, the most effective educational strategies are likely to integrate simulation and traditional methods in a purposeful sequence aligned with specific learning objectives.

### 3.10 Applications across disciplines: summary points

- Overall, simulation-based tools are widely used across veterinary disciplines, with the strongest representation in veterinary medicine education (undergraduates).
- The canine was the species with most targeted models/simulators created.
- Clinical skills, Surgery and Anatomy are the areas where models are more extended and show most effectiveness.
- Simulation methods generally match or outperform traditional teaching for short-term gains in knowledge, procedural accuracy, and confidence, especially in anatomy and basic clinical or procedural skills, while traditional methods remain essential for developing clinical reasoning, judgement, and adaptability.

Evidence consistently supports their value for skill acquisition, particularly in anatomy, basic clinical procedures, surgery, and emergency care. However, the extent and quality of evidence vary markedly between disciplines, and direct comparisons with traditional teaching methods remain uneven. The authors consider that simulation is currently most effective in disciplines where skills are procedural, repeatable, and high risk, whereas its role in more complex, integrative clinical decision-making remains underdeveloped and warrants further investigation.

## 4 Impact on learning and skill acquisition

This section primarily addresses Objective 2 and Objective 3 by synthesizing evidence on the educational impact of simulation-based training in veterinary education. It evaluates outcomes across psychomotor, cognitive, and affective domains and considers how simulators support assessment, feedback, and competency-based learning, including comparisons with traditional instructional methods where evidence allows.

### 4.1 Psychomotor skills development

Psychomotor skills are movement-oriented activities that require the integration of motor coordination with cognitive processing, forming the foundation of many clinical and diagnostic procedures in veterinary and medical education. These skills are best acquired through repetitive, low-risk practice, where simulation-based training provides a pivotal advantage by allowing learners to refine techniques without compromising patient safety (23, 165). In a study by Smeak et al. (133) it was demonstrated that students exhibited improved accuracy and confidence following structured simulation practice, underscoring the role of controlled repetition in skill acquisition. Grevemeyer et al. (87) corroborated these findings, reporting that 95% of students at Ross University perceived advancements in their psychomotor skills through engagement in clinical skills laboratories, highlighting the effectiveness of hands-on experimental learning environments. Furthermore, Garcia-Ara et al. (85) conducted a meta-analysis of multiple studies supporting simulator-based training as pedagogical tool. These findings were reinforced by studies showing significant improvements in knowledge acquisition, time efficiency, procedural accuracy, enhancement of spatial understanding and retention of information, when models were used (46, 47, 124, 153). From the articles evaluated in this review, 75/101 were focused on psychomotor skills. Thirty-seven were models and 38 simulators. There was a similar proportion between fidelity and behavior. This is an interesting result as it does not show the priority of a dynamic teaching tool over static representations.

### 4.2 Cognitive and affective learning outcomes

Cognitive and affective learning outcomes represent two fundamental domains of educational achievement. Cognitive outcomes refer to the acquisition and application of knowledge. In contrast, affective outcomes refer to the development of attitudes, values, motivations, and emotional responses of learners’ toward knowledge. Together with psychomotor outcomes (hands-on skills), they form a holistic model of competence in health sciences education (23).

In terms of cognitive and affective outcomes, simulation learning significantly boosts student engagement and retention of information (43). Baillie et al. (27) highlighted that simulations provide a dynamic environment conducive to enhancing self-efficacy and reducing anxiety regarding real-life clinical scenarios. In their innovative study on the “Equine Virtual Farm,” the authors created and immersing virtual environment reflecting how problems are encountered in real life. Their aim was to integrate both physiological knowledge and laboratory skills. Results from the study showed that cognitive engagement was further underscored via brain imaging techniques, showing the engagement of problem-solving regions when participating in simulation games (166).

The role of VR and AR cannot be overstated; studies illustrated that even with minimal prior experience, participants found VR modules enriching, thereby enhancing the educational experience (167).

Thus, the trajectory of simulation in veterinary medicine suggests a strong shift toward incorporating multifaceted, technology-enhanced resources that transcend conventional training methodologies. With consistent findings indicating that students favor interactive, gamified learning environments, educators are encouraged to consider flexible, engaging modalities that blend traditional with modern pedagogies. Interactive learning through gamification highlights the potential benefits of these methods in promoting engagement and knowledge retention (131, 168). In contrast to psychomotor skills, only 23/101 studies were focused on knowledge acquisition and 3/101 had both knowledge and psychomotor aims. From the ones focusing on knowledge acquisition, there was similar distribution between models and simulators types, showing there was not a clear preference inside this group.

### 4.3 Assessment and feedback mechanisms

Simulators also provide an advantageous platform for objective assessment through real-time performance metrics (28). These metrics bolster competency-based learning, as they enable immediate feedback that is crucial for skill refinement and self-assessment. Also, it reduces the need for supervised training and provides opportunities for self-directed learning and peer feedback when groups are used (131). For example, the integration of customizable assessment tools in simulators allows institutions to evaluate a student’s progress comprehensively and emphasizes the importance of regular feedback in improving student performance and overall learning experience (85).

Studies suggest that adaptive learning platforms are pivotal in personalizing training experiences, offering tailored content that addresses individual gaps in learning (59). This integration is vital, especially considering the increasing student population in veterinary programs, which has led to limited practical skill development opportunities.

From the original articles included in this review 44/101 had both an objective assessment method and a questionnaire to know the participants opinion. In 40/101 feedback was extracted only from a questionnaire and in 17/101 results were extracted from objective methods only. Participants considered the use of models and simulators a good learning experience in all the studies, despite their outcome. These results show a lack of systematical objective assessment when studying these tools of teaching purposes and an overuse of subjective assessment to evaluate conclusions.

### 4.4 Impact on learning: summary points

- Simulation-based training enhances psychomotor skill development, with students showing improved accuracy, confidence, and procedural performance through repetitive, low-risk practice.
- Cognitive and affective learning outcomes also benefit from simulation, as interactive, environments increase engagement, self-efficacy, and knowledge retention, particularly in gamified or immersive scenarios.
- Across reviewed studies, students consistently rated simulators and models as valuable learning tools. However, objective assessment and feedback should be improved in future research and is currently missing in a large proportion of studies.

Taken together, the evidence suggests that simulation-based training is an effective approach for developing psychomotor skills, enhancing cognitive understanding, and positively influencing learner confidence and engagement. In many contexts, simulation performs at least as well as traditional teaching methods for short-term learning outcomes, while offering additional pedagogical advantages such as standardized exposure and immediate feedback. A number of articles only describing model fabrication or expert self-assessment were not included for analysis in the review as there was no learning impact evaluation. In the authors’ opinion, the strongest educational value of simulation lies in early and intermediate stages of training, where deliberate practice and error tolerance are critical, rather than as a replacement for advanced clinical experience with live patients.

## 5 Challenges and limitations

This section addresses Objective 3 by critically examining the limitations associated with the use of physical models and simulators in veterinary education. It highlights financial, technical, pedagogical, and evidentiary barriers that influence the adoption, implementation, and effectiveness of simulation-based training.

The use of simulators in veterinary education presents numerous challenges and limitations that can hinder their widespread adoption and effectiveness. Many of the challenges identified in the literature—particularly cost, limited realism, and accessibility—underscore the importance of principled model selection, as outlined in Section 2.3.

### 5.1 Ethical considerations in simulation training

From an ethical standpoint, the use of models and simulations can both benefit students and animal’s welfare. The use of these techniques can reduce students stress by creating a safe environment of learning (24). Moreover, these models align well with the 3Rs principle—Replacement, Reduction, and Refinement of animal use in education. The use of cadavers and live animals for teaching present a range of ethical, logistical, and pedagogical challenges (3–8). In response to these, the 3R principle promotes humane education practices and ensures that students are trained in a manner that respects animal welfare (169–171). The authors believe these considerations should be reviewed alongside educational effectiveness to ensure that student competency and animal welfare are advanced concurrently.

### 5.2 Financial barriers

High-fidelity simulators are often prohibitively expensive, creating significant financial obstacles for veterinary education programs, particularly in low-resource settings (20). The authors were surprised by the low number of papers specifying the actual cost of the model or simulator described, and more transparency will be needed in the future to compare and implement these teaching tools. As highlighted by Hunt et al., the costs associated with purchasing, maintaining, and updating simulators restricts access to essential training tools for many institutions (20). Moreover, the necessity of trained personnel to operate simulators and facilitate their use further increases operational costs, potentially diverting funds from other crucial educational initiatives (20). The implementation of synthetic materials and 3Dp technology into simulator model design has helped decrease this gap. However, the technology needed to create those simulators can also be expensive and most of them have a single utility, resulting in a need for multiple simulators for different purposes. Additionally, the maintenance of affordable models can be time consuming, as they usually need frequent changes in their structure to maintain their function.

### 5.3 Realism and transferability

While simulators aim to replicate clinical situations, they frequently fall short in terms of tactile realism and emotional engagement, which can adversely affect the transferability of skills learned to real-life clinical settings. Such models may have significant differences in texture, fragility and flexibility compared to real tissue, which may result in a less translatable tissue handling technique. In their study, Braid et al., indicates that the lack of authentic sensory feedback in some simulation scenarios can lead to students developing inadequate skills when transitioning from simulated environments to actual animal handling and surgery (19). This dissonance between simulation and real-world application may lead to a gap in practitioners’ confidence and their ability to perform effectively under clinical pressures (172). Hence, simulators are a good first environment to train skills, but further research will be needed to investigate the transferability of skills and most likely, will never completely replace live animal clinical scenarios.

### 5.4 Versatility and resilience

While commercially available simulators can increase the accessibility of these products, they are often designed with a narrow focus. Usually, each simulator is targeting a specific skill and may therefore offer limited versatility beyond its intended purpose. This single-function design, while useful for targeted training, can restrict broader educational utility, as illustrated in a report detailing a low-cost training program that relied on multiple specialized models (173). Consequently, the volume of simulators required to comprehensively replace live animal or patient-based training becomes logistically and financially burdensome. To address these challenges, the development and integration of low-cost models and or multipurpose simulators, capable of supporting a range of clinical scenarios and skill sets has become popular in veterinary education. However, these low-cost models usually are not as resilient as the commercially available and need maintenance or remaking of the model after multiple uses (34).

### 5.5 Multidisciplinary approach

In addition, the creation of these simulators necessitates a multidisciplinary approach, involving collaborations between veterinarians and engineers of different kinds, not only to create the models, but also to keep them updated. This relationship may not be available in all institutions and requires time and effort that can complicate the implementation and creation of new simulators.

### 5.6 Faculty and student training

Effective integration of simulation into veterinary curricula requires instructors who are not only skilled in their subject matter but also trained in pedagogical methods specific to simulation-based learning. Without proper training, instructors may struggle to facilitate learning effectively, thereby diminishing the potential benefits that simulators can offer in skill acquisition and clinical preparedness. Depending on the model used, training may need to be continued or repeated to keep up with updates and technological advancements.

Moreover, students need to be trained in the technology and software specific to the simulator, in addition to the practical skill that the model is intended for. Proficiency in these digital tools is essential to fully engage with the simulation models and to optimize the learning of the intended medical skills.

### 5.7 Cybersickness

Virtual and augmented reality-based simulations may also be associated with physical symptoms in some users. While most people appear to adjust to VR simulators without problems, reported adverse effects with VR use include motion sickness, dizziness, headache, sleepiness (37, 174). In a review by Simón-Vicente et al., the most commonly reported adverse effects were nausea, disorientation, and oculomotor disturbances such as blurred vision, headache, eyestrain (174).

### 5.8 Challenges and limitations: summary points

- Ethical considerations should be reviewed alongside educational effectiveness to ensure that student competency and animal welfare are advanced concurrently.
- There is lack of longitudinal data across studies, that highlights the limitations of these teaching tools, including the lack of evidence on long-term competency, patient outcomes and model or simulator costs.
- Realism and transferability remain limited, as simulators often lack authentic tactile and emotional fidelity, reducing the reliability of transferring skills to real clinical settings.
- Versatility and durability are constrained, with many simulators designed for single skills, making large collections costly and low-cost alternatives less resilient over repeated use.
- Model/Simulator development requires multidisciplinary collaboration, which can be difficult for institutions lacking engineering partnerships or the resources to maintain and update models.
- Effective use depends on faculty and student training, as both groups must learn simulation-specific pedagogy and technology; VR-based tools may also cause cybersickness in some users.

Despite the growing body of literature on model and simulation-based training in veterinary education, the available scientific evidence presents several important limitations that constrain the conclusions of this review. Many studies focus on short-term outcomes such as immediate skill performance, knowledge retention, or learner perceptions, with relatively few investigations assessing long-term skill retention, transferability to live clinical settings, or professional competency following graduation. Study designs are frequently heterogeneous, employing varied outcome measures, small sample sizes, and discipline-specific assessment tools, which limits comparability across studies and restricts opportunities for meta-analysis. Additionally, direct side-by-side comparisons between simulation-based and traditional teaching methods remain unevenly distributed across disciplines, with stronger evidence in procedural and technical skills than in complex clinical decision-making or integrative practice. Finally, a number of publications do not employ standardised terminology or objective assessment methods. In the authors’ view, these gaps represent the most significant barrier to fully achieving the review’s objectives, particularly with respect to evaluating long-term educational impact and clinical relevance. Addressing these limitations will require coordinated, multi-institutional research efforts using standardized outcome measures and longitudinal study designs capable of capturing competency development over time.

In conclusion, while models and simulators hold great promise for enhancing veterinary education, addressing the associated challenges—financial constraints, realism issues, faculty training deficits, and the need for more research—is vital for their successful implementation and integration into veterinary curricula. In the authors’ view, this absence of long-term outcome data represents the most critical limitation of current simulation research and restricts the extent to which simulation-based training can be confidently positioned as a substitute for traditional clinical exposure. Addressing this gap should be a priority for future research.

## 6 Future perspectives

This section addresses Objective 4 by considering emerging technologies and future directions for simulation-based veterinary education. It incorporates the authors’ interpretations of current trends and outlines expectations for how simulation may evolve across different areas of veterinary practice and training.

### 6.1 Technology innovations

In this constantly evolving world, there are rapid changes in the technology available, and there is therefore a need to adapt to new tools and teaching opportunities. Some of them are already emerging, like the use of 3Dp with biological materials. This has particularly gained attention for its potential in personalized medicine, allowing for the production of organ or tissue models tailored to individual patient needs. 3Dp with biological materials may provide more realistic teaching models, particularly with regards to tissue manipulation in surgical training.

This technology is quickly followed by 4D printing (4Dp). 4D printing is a 3Dp process that can change the shape or properties of materials over time and in a specific environment (175). This technology differs from traditional 3Dp in that the printed materials can respond dynamically to environmental forces or changes, such as humidity or temperature. This modification presents opportunities for developing dynamic training models that better mimic the complexities of living anatomy (175). It includes artificial organs or prosthetics that can be modified to reflect animal’s growth or changes in different physical condition. For example, a wound healing or injury treatment that evolutions without having to change the material each time (176, 177).

The integration of AI into surgical simulators further enhances their educational value. It may also be useful in diagnostic imaging or any clinical scenario. AI-driven systems can simulate physiological responses, provide clinical scenarios or adapt scenarios based on student performance, and provide personalized feedback, thereby supporting individualized learning pathways and improving surgical competency (52, 83).

Meanwhile, the use of VR for immersive training experiences allows students to visualize simulated surgical environments layered with real-time feedback and information, significantly enhancing their understanding of intricate procedures (37). In the future, it may also be adapted for use in other clinical skills, such as teaching of minimally invasive procedures, practicing response to emergency situations, practicing anesthetic induction and maintenance.

### 6.2 Curriculum standardization

To fully harness the potential of these technologies, veterinary curricula must evolve toward greater standardization. The integration of simulation-based tools, AI modules, and AR platforms requires structured pedagogical frameworks that ensure consistency across institutions. Standardized curricula facilitate benchmarking, accreditation, and the development of competency-based assessments, aligning educational outcomes with technological capabilities. Moreover, curriculum standardization supports faculty development, enabling educators to effectively implement and evaluate advanced training tools.

### 6.3 Global equity and interdisciplinary learning

While technological innovation offers transformative potential, equitable access remains a critical concern. Institutions with limited resources may struggle to adopt high-fidelity simulators or advanced printing technologies. To address this, collaborative platforms for sharing 3D printed models and simulation protocols have emerged, fostering cross-institutional exchange and democratizing access to high-quality educational resources (92, 178). These networks promote innovation and best practices, ensuring that veterinary students worldwide benefit from cutting-edge training tools. Collectively, these advancements signal a new era in veterinary education that emphasizes practical, hands-on learning supported by cutting-edge technology and collaboration.

Furthermore, interdisciplinary collaboration—bridging veterinary science with fields such as biomedical engineering, computer science, and materials science—accelerates the development of novel educational technologies. Such partnerships enrich the design and functionality of simulators, enhance realism, and expand the scope of training applications. Collectively, these efforts signal a new era in veterinary education, one that emphasizes practical, hands-on learning supported by technological sophistication, ethical responsibility, and global inclusivity.

### 6.4 Future perspectives: summary points

- 4Dp should be considered as an emergent tool to create simulators.
- AI will likely shape the future of teaching.
- Curriculum standardization is needed to ensure consistency across institutions, as well as an effort towards global equity regarding access to these teaching tools.

Future developments in simulation are likely to be driven by advances in three- and four-dimensional printing, artificial intelligence, and immersive virtual and augmented reality technologies. The authors anticipate that these innovations will increasingly support personalized, adaptive learning and enhance the realism of simulated clinical scenarios. However, they also expect that models and simulator will remain a complementary rather than replacement strategy, with its greatest impact achieved when guided by clear educational objectives, integrated thoughtfully within standardized curricula and supported by interdisciplinary collaboration and robust educational research.

## 7 Conclusion

Physical models and simulation-based tools now represent an established and rapidly evolving component of veterinary education. This review demonstrates that a wide spectrum of models and simulators is currently available, ranging from low-fidelity synthetic trainers to highly immersive virtual, augmented, and mixed reality platforms. These tools can be meaningfully classified according to fidelity, complexity, and behavior. However, a practical framework for educators when selecting resources aligned with specific learning objectives is still missing.

Across veterinary disciplines, the existing evidence indicates that simulation-based training is an effective educational strategy, particularly for anatomy, basic clinical procedures, selected surgical skills, diagnostic imaging, and emergency interventions such as cardiopulmonary resuscitation. When compared with traditional teaching methods, simulation commonly achieves equivalent or superior short-term learning outcomes, while offering additional benefits including reduced student anxiety, improved confidence, standardized exposure to clinical scenarios, and enhanced opportunities for deliberate practice and feedback. However, the strength of comparative evidence varies considerably between disciplines, and direct side-by-side evaluations using standardized outcome measures remain limited.

Despite these advantages, significant challenges persist. Financial constraints, variable realism, faculty and student training requirements, and restricted access to high-fidelity technologies continue to influence the feasibility and scalability of simulation-based education. Importantly, this review highlights a substantial gap in longitudinal evidence assessing the transferability of simulated skills to real clinical performance and long-term professional competence. As a result, while simulation clearly supports early skill acquisition and preparedness, its capacity to predict or replace clinical proficiency gained through live-animal experience remains insufficiently defined.

Looking ahead, the authors anticipate that these teaching to old will continue to expand as a complementary, rather than substitutive, approach to traditional veterinary training. Advances in 3Dp and 4Dp, AI–driven adaptive learning systems, and increasingly immersive VR and AR platforms are likely to enhance realism, personalization, and accessibility. At the same time, greater curriculum standardization, interdisciplinary collaboration, and multi-institutional research efforts will be essential to ensure equitable access and robust evaluation of educational outcomes.

In conclusion, model and simulation-based training aligns ethical responsibility with pedagogical innovation and offers clear benefits for veterinary education. Its greatest value lies in providing a structured, low-risk environment in which learners can develop foundational skills and confidence before entering clinical practice. Continued research focusing on long-term outcomes, clinical transferability, and cost-effectiveness will be critical to defining the optimal role of simulation in preparing future veterinarians for the complex demands of professional practice.
