# Supplementary material for: Physical models and simulators in veterinary education: current status, learning impact, and future perspectives
Source: Front Vet Sci. 2026 Apr 17;13:1774849. doi: 10.3389/fvets.2026.1774849 (PMC13133729; doi:10.3389/fvets.2026.1774849)
Supplement: Supplementary file 1 [file Table_1.docx]

| **Article title** | **Authors** | **Journal** | **Year** | **Country of first author’s affiliation** | **Model or Simulator** | **Fidelity** | **Complexity** | **Behavior** | **Student target** | **Compared to** | **Outcome assessment method** | **Results vs comparator** | **Learning objective** | **Clinical topic** | **Target species** |
| --- | --- | --- | --- | --- | --- | --- | --- | --- | --- | --- | --- | --- | --- | --- | --- |
| Evaluation of video tape and a simulator for instruction of basic surgical skills | Smeak et al.(1) | Vet surgery | 1991 | USA | Model | Low | Syntetic simple | Static | Undergraduate | Traditional | Objective | Better | Psychomotor skill | Surgery | Dog |
| Evaluation of an autotutorial-simulator program for instruction of hollow organ closure | Smeak et al.(2) | Vet surgery | 1994 | USA | Model | Low | Syntetic simple | Static | Undergraduate | Traditional | Objective + questionnaire | Worse | Psychomotor skill | Surgery | Mixed |
| Evaluation of a hemostasis model for teaching basic surgical skills | Olsen et al. (3) | Vet surgery | 1996 | USA | Model | Low | Syntetic simple | Static | Undergraduate | Traditional | Questionnarie | Same | Psychomotor skill | Surgery | Dog |
| Evaluation of a hemostasis model for teaching ovariohysterectomy in veterinary surgery | Griffon et al. (4) | Vet surgery | 2000 | UK | Model | Low | Syntetic simple | Static | Undergraduate | Traditional / cadaver | Objective | Better | Psychomotor skill | Surgery | Dog |
| Preliminary development and evaluation of a bovine rectal palpation simulator for training veterinary students | Baillie et al. (5) | cattle practice | 2003 | UK | Simulator | Hybrid | Mixed | Dynamic | Undergraduate | Traditional (live cows) | Questionnarie | N/A | Psychomotor skill | Clinical skills | Cow |
| Integrating a Bovine Rectal Palpation Simulator into an Undergraduate Veterinary Curriculum | Baillie et al. (6) | JVME | 2005 | UK | Simulator | Hybrid | VR | Dynamic | Undergraduate | N/A | Questionnarie | N/A | Psychomotor skill | Rectal palpation | Cow |
| Evaluation of a dog abdominal surrogate model for teaching basic surgical skills | Mori et al. (7) | J Jpn Vet Med Assoc | 2006 | Japan | Model | Low | Syntetic simple | Static | Undergraduate | None | Questionnarie | N/A | Psychomotor skill | Surgery | Mixed |
| Teaching Surgery to the Veterinary Novice: The Ohio State University Experience | Smeak (8) | JVME | 2007 | US | Model | Low | Synthetic simple | Static | Undergraduate | None | Objective + questionnarie | Same | Knowledge+ physco | Surgery | Dog and cat |
| Teaching Transrectal Palpation of the Internal Genital Organs in Cattle | Bossaert et al. (9) | JVME | 2009 | Belgium | Model | Low | Synthetic simple | Static | Undergraduate | Traditional (live cows) | Objective + questionnaire | Same | Psychomotor skill | Clinical skills | Cow |
| Teaching Bovine Abdominal Anatomy: Use of a haptic simulator | Kinnison et al. (10) | Anatomical science education | 2009 | UK | Simulator | Hybrid | Mixed | Dynamic | Undergraduate | None | Questionnarie | N/A | Knowledge | Anatomy | Cow |
| Assessment of laparoscopic skills before and after simulation training with a canine abdominal model | Fransson and Ragle (11) | JAVMA | 2010 | USA | Model | Low | Syntetic simple | Static | Undergraduate | Traditional | Objective | Same/better | Psychomotor skill | Surgery | Dog |
| Validation of the educational potential of a simulator to develop abilities and skills for the creation and maintenance of an intravenous cannula | Perez-Rivero et al. (12) | Animal Research | 2011 | Mexico | Simulator | Low | Syntetic simple | Dynamic | Undergraduate | Traditional | Objective | Better | Psychomotor skill | Clinical skills | Mixed |
| Development and evaluation of a high-fidelity canine patient simulator for veterinary clinical training | Fletcher et al. (13) | JVME | 2012 | USA | Simulator | High | Mixed | Dynamic | Undergraduate | None | Questionnarie | N/A | Psychomotor skill | CPR | Dog |
| Evaluation of a dental model for training veterinary students | Lumbis et al. (14) | JVME | 2012 | UK | Model | Low | Synthetic simple | Static | Undergraduate | Traditional | Objective + questionnarie | Same | Psychomotor skill | Clinical skills | Dog |
| Augmented reality intravenous injection simulator based 3D medical imaging for veterinary medicine. | Lee et al. (15) | Vet J | 2012 | Korea | Simulator | Hybrid | AR | Dynamic | Undergraduate | Traditional | Objective + questionnaire | Better | Psychomotor skill | Clinical skills | Dog |
| Development and validation of an equine nerve block simulator to supplement practical skills training in undergraduate veterinary students | Gunning et al. (16) | Veterinary record | 2013 | UK | Simulator | High | Synthetic simple | Dynamic | Undergraduate | Traditional / cadaver | Objective + questionnarie | Same | Knowledge+ physco | Anaesthesia | Horse |
| Evaluation of a training model to teach veterinary students a technique for injecting the jugular vein in horses | Eichel et al. (17) | JVME | 2013 | Germany | Simulator | Low | Synthetic simple | Dynamic | Undergraduate | Traditional | Objective | Better | Psychomotor skill | Clinical skills | Horse |
| “Let’s Get Physical”: Advantages of a Physical Model Over block simulator to supplement practical skills training in undergraduate veterinary students | Preece et al. (18) | Anatomical science education | 2013 | UK | Model | Low | 3D | Static | Undergraduate | Traditional | Objective + questionnaire | Better book, worse real | Knowledge | Anatomy | Horse |
| Design and validation of a simulator for equine joint injections | Fox et al. (19) | JVME | 2013 | UK | Simulator | Low | Syntetic simple | Dynamic | Undergraduate | Traditional / cadaver | Questionnarie | Better + Same | Psychomotor skill | Clinical skills | Horse |
| Development and Evaluation of a Canine Laparoscopic Simulator for Veterinary clinical Teaching | Usón-Gargallo et al. (20) | JVME | 2014 | Spain | Simulator | Hybrid | Mixed | Dynamic | Postgraduate | None | Objective + questionnarie | N/A | Psychomotor skills | Surgery | Dog |
| Validation of a realistic simulator for veterinary gastrointestinal endoscopy training | Usón-Gargallo et al. (21) | JVME | 2014 | Spain | simulator | Hybrid | Syntetic simple | Dynamic | Under + post | None (novice vs experts) | Objective + questionnaire | N/A | Psychomotor skill | Clinical skills | Dog |
| Construct validation of a small-animal thoracocentesis simulator | Williamson et al. (22) | JVME | 2014 | West Indies | Model | Low | Syntetic simple | Static | Under + post | None | Questionnarie | N/A | Psychomotor skill | Clinical skills | Mixed |
| Development of a training model for small animal thoracocentesis and chest tube thoracostomy | Williamson and Rito (23) | ATLA | 2014 | West Indies | Model | Low | Syntetic simple | Static | Undergraduate | None | Questionnarie | N/A | Psychomotor skill | Clinical skills | Mixed |
| Canine prostate palpation simulator as a teaching tool in veterinary education | Capilé et al. (24) | JVME | 2015 | Brazil | Model | Low | Synthetic simple | Static | Undergraduate | None | Questionnarie | Better | Psychomotor skill | Clinical skills | Dog |
| Validation of an effective, easy-to-make hemostasis simulator | Guisto et al. (25) | JVME | 2015 | Italy | Simulator | Low | Syntetic simple | Dynamic | Undergraduate | Traditional / cadaver | Objective | Better | Psychomotor skill | Surgery | Horse |
| Development and validation of a feline abdominal palpation model and scoring rubric | Williamson et al. (26) | Veterinary record | 2015 | USA | Model | Low | Syntetic simple | Static | Under + post | None (novice vs experts) | Objective | Same | Psychomotor skill | Anatomy | Cat |
| Stress Response of Veterinary Students to Gynaecological Examination of Horse Mares – Eﬀects of Simulator-Based and Animal-Based Training | Nagel et al. (27) | Reproduction in domestic animals | 2015 | Austria | Simulator | Hybrid | VR | Static | Undergraduate | Traditional (live) | Objective | worse | Knowledge | Anatomy | Horse |
| Validation of a model for teaching canine fundoscopy | Nibblett et al. (28) | JVME | 2015 | USA | Model | Low | Syntetic simple | Static | Undergraduate | Traditional (live) | Objective + questionnaire | Same | Psychomotor skill | Clinical skills | Dog |
| Development and efficacy of a canine pelvic limb model used to teach the cranial drawer and tibial compression tests in the stifle joint | Troy and Bergh (29) | JVME | 2015 | USA | Model | Low | Syntetic simple | Dynamic | Undergraduate | none | Questionnarie | N/A | Knowledge | Clinical skills | Dog |
| Development and evaluation of two canine low-fidelity simulation models. | Aulmann et al. (30) | JVME | 2015 | Germany | Model | Low | Syntetic simple | Static | Undergraduate | Traditional | Objective | Better | Psychomotor skill | Clinical skills | Dog and cat |
| Assessment of Laparoscopic Skills in Veterinarians Using a Canine Laparoscopic Simulator | Tapia-Araya et al. (31) | JVME | 2016 | Spain | Simulator | Low | Synthetic simple | Dynamic | Postgraduate | None | Objective + questionnarie | N/A | Psychomotor skills | Surgery | Dog |
| Efficacy of teaching the Gambee suture pattern using simulated versus cadaveric small intestine | Caston et al. (32) | Vet surgery | 2016 | USA | Model | Low | Syntetic simple | Static | Undergraduate | Traditional / cadaver | Objective | Same | Psychomotor skill | Surgery | Horse |
| The SimSpay—student perceptions of a low-cost build-it-yourself model for novice training of surgical skills in canine ovariohysterectomy | Langebæk et al. (33) | JVME | 2016 | Denmark | Model | Low | Syntetic simple | Static | Undergraduate | None | Questionnarie | N/A | Psychomotor skill | Surgery | Dog and cat |
| Evaluation of veterinary student surgical skills preparation for ovariohysterectomy using simulators: a pilot study | Read et al. (34) | JVME | 2016 | Canada | Model | Low | Syntetic simple | Static | Undergraduate | Model + Video | Questionnarie | Better + Same | Psychomotor skill | Surgery | Dog |
| Training Veterinary Students to Perform Ovariectomy Using theMOOSE Spay Model with Traditional Method versus the Dowling Spay Retractor | Fahie et al. (35) | JVME | 2016 | USA | Model | Low | Syntetic simple | Static | Undergraduate | Model | Objective + questionnaire | Same | Psychomotor skill | Surgery | Dog and cat |
| Veterinary student confidence after practicing with a new surgical training model | Badman et al. (36) | JVME | 2016 | Sweeden | Model | Low | Syntetic simple | Static | Undergraduate | Traditional | Questionnarie | Better | Psychomotor skill | Surgery | Dog |
| Comparison between training models to teach veterinary medical students basic laparoscopic skills | Levi et al. (37) | JVME | 2016 | USA | Simulator | Hybrid | Mixed | Dynamic | Undergraduate | Simulator | Objective + questionnaire | Same | Psychomotor skill | Surgery | Mixed |
| Student attitudes regarding the educational value and welfare implications in the use of model eyes and live dogs in teaching practical fundus examination | Williams et al. (38) | Open Vet J | 2016 | UK | Model | Low | Syntetic simple | Static | Undergraduate | Traditional / cadaver | Questionnarie | Same/worse | Knowledge | Clinical skills | Dog |
| Value of artisanal simulators to train veterinary students in performing invasive ultrasound-guided procedures | Hage et al. (39) | Adv physiol educ | 2016 | Brazil | Model | Low | Syntetic simple | Static | Under + post | None | Questionnarie | N/A | Psychomotor skill | Clinical skills | Mixed |
| Preliminary evaluation of learning performance of the simplest bovine trans-rectal palpation phantom for training veterinary students | Zolhavarieh et al. (40) | IRANIAN JOURNAL OF RUMINANTS HEALTH RESEARCH | 2016 | Iran | Simulator | Low | Syntetic simple | Dynamic | Under + post | None | Questionnarie | N/A | Psychomotor skill | Clinical skills | Cow |
| Development and validation of a model for training equine phlebotomy and intramuscular injection skills | Williamson et al. (41) | JVME | 2016 | USA | Simulator | Low | Syntetic simple | Dynamic | Undergraduate | Traditional / cadaver | Objective | Same | Psychomotor skill | Clinical skills | Horse |
| An easy to produce and economical three-dimensional brain phantom for stereotactic computed tomographic-guided brain biopsy training in the dog | Sidhu et al. (42) | Vet surgery | 2017 | USA | Model | Low | Syntetic simple | Static | Postgraduate | None (novice vs experts) | Objective | N/A | Psychomotor skill | Surgery | Dog |
| Technical skills training for veterinary students: A comparison of simulators and video for teaching standardized cardiac dissection | Allavena et al. (43) | JVME | 2017 | Australia | Model | Low | Syntetic simple | Static | Undergraduate | Video | Objective + questionnaire | Same | Knowledge | Anatomy | Horse |
| Design and validation of a three-dimensional printed flexible canine otoscopy teaching model | Nibblett et al. (44) | Empirical investigations | 2017 | USA | Model | Low | Synthetic 3d | Static | Undergraduate | None | Questionnarie | Same | Psychomotor skill | Clinical skills | Dog |
| The value of homemade phantoms for training veterinary students in the ultrasonographic detection of radiolucent foreign bodies | Mariano Beraldo et al. (45) | Adv Physiol Educ | 2017 | Brazil | Model | Low | Syntetic simple | Static | Undergraduate | None | Questionnarie | N/A | Psychomotor skill | Diagnostic imaging | Mixed |
| Computer Assisted Learning: Assessment of the Veterinary Virtual Anatomy Education Software IVALA^TM^ | Little et al. (46) | Veterinary science | 2018 | US | Model | High | VR | Static | Undergraduate | Traditional / cadaver | Objective + questionnarie | Better | Knowledge | Anatomy | Dog |
| Proof of concept of a workflow methodology for the creation of basic canine head anatomy veterinary education tool using augmented reality | Christ et al. (47) | Plos one | 2018 | UK | Simulator | High | AR | Dynamic | Undergraduate | None | Questionnarie | Same | Knowledge | Anatomy | Dog |
| Face, construct, and concurrent validity of a simulation model for laparoscopic ovariectomy in standing horses | Elarbi et al. (48) | JAVMA | 2018 | USA | Simulator | Low | Synthetic simple | Dynamic | Undergraduate | None | Objective + questionnarie | N/A | Psychomotor skills | Surgery | Horse |
| Comparison of the use of fresh-frozen canine cadavers and a realistic composite ex vivo simulator for training in small animal flexible gastrointestinal endoscopy | Pérez-Merino et al. (49) | JAVMA | 2018 | Spain | Simulator | Hybrid | Synthetic simple | Dynamic | Undergraduate | Traditional | Objective + questionnarie | Same | Psychomotor skills | Clinical skills | Dog |
| Production of Accurate Skeletal Models of Domestic Animals Using Three-Dimensional Scanning and Printing Technology | Li et al. (50) | anatomical science education | 2018 | China | Model | Low | 3D | Static | Undergraduate | Traditional | Objective + questionnaire | Same | Knowledge | Anatomy | Cow |
| Survey of clinician and student impressions of a synthetic canine model for gastrointestinal surgery training | Au Yong et al. (51) | Vet surgery | 2018 | USA | Simulator | High | Syntetic simple | Static | Undergraduate | Traditional | Questionnarie | Better | Psychomotor skill | Surgery | Dog |
| Introduction and assessment of an inanimate model for basic surgical skills training of veterinary students | Mehrdadi and Marjani (52) | Journal Surgical simulator | 2018 | Iran | Model | Low | Syntetic simple | Static | Undergraduate | Traditional (live) | Objective | Better | Psychomotor skill | Surgery | Dog |
| Development and validation of a low-fidelity, low-cost surgical simulation model to teach canine orchiectomy | Motta et al. (53) | Clinical Therionegoloy | 2018 | USA | Model | Low | Syntetic simple | Static | Undergraduate | Traditional | Objective + questionnaire | Better | Psychomotor skill | Surgery | Dog |
| Validation of a model of feline ureteral obstruction as a tool for teaching microsurgery to veterinary surgeons | Phillips et al. (54) | Vet surgery | 2018 | USA | Model | Low | Syntetic simple | Static | Postgraduate | None (novice vs experts) | Questionnarie | Same | Psychomotor skill | Surgery | Cat |
| Development of veterinary anesthesia simulations for pre-clinical training | Jones et al. (55) | JVME | 2018 | USA | Simulator | High | VR | Dynamic | Undergraduate | None | Questionnarie | Same/better | Psychomotor skill | Clinical skills | Mixed |
| Development and student evaluation of an anatomically correct high-fidelity calf leg model | French et al. (56) | JVME | 2018 | West Indies | Model | Low | Synthetic 3d | Static | Undergraduate | Model | Questionnarie | Better | Psychomotor skill | Clinical skills | Cow |
| Training method and other factors affecting student accuracy in bovine pregnancy diagnosis | Annandale et al. (57) | JVME | 2018 | South Africa | Simulator | Low | Syntetic simple | Dynamic | Undergraduate | Traditional (live cows) | Questionnarie | Better/worse | Psychomotor skill | Clinical skills | Cow |
| Evaluation of surgical models for training veterinary students to perform enterotomies | Grimes et al. (58) | Veterinary Surgery. | 2019 | US | Model | High | Synthetic simple | Static | Under + post | Traditional / cadaver | Questionnarie | Same | Psychomotor skills | Surgery | Mixed |
| Use of Three-Dimensional Printing Models for Veterinary Medical Education: Impact on Learning How to Identify Canine Vertebral Fractures | Suñol et al. (59) | JVME | 2019 | Spain | Model | Low | Synthetic 3d | Static | Undergraduate | Traditional | Objective + questionnarie | Better + Same | Knowledge | Anatomy | Dog |
| IVET, an Interactive Veterinary Education Tool | Xiberta and Boada (60) | J animal scien | 2019 | Spain | Simulator | High | VR | Dynamic | Undergraduate | None | Objective + questionnarie | Same | Knowledge | Anatomy | Mixed |
| Development and evaluation of a high-fidelity canine laparoscopic ovariectomy model for surgical simulation training and testing | Chen et al. (61) | JAVMA | 2019 | USA | Simulator | Low | Synthetic simple | Dynamic | Undergraduate | Commerical model | Objective + questionnarie | Same | Psychomotor skills | Surgery | Dog |
| Survey of instructor and student impressions of a high-fidelity model in canine ovariohysterectomy surgical training | Au Yong et al. (62) | Vet surgery | 2019 | USA | Simulator | High | Syntetic simple | Static | Undergraduate | Traditional | Objective + questionnaire | Same/better | Psychomotor skill | Surgery | Dog |
| Comparison of 2 canine celiotomy closure models for training novice veterinary students | Williamon et al. (63) | Vet surgery | 2019 | USA | Model | Low | Syntetic simple | Static | Postgraduate | Model | Questionnarie | Same | Psychomotor skills | Surgery | Dog |
| Evaluating veterinary student skill acquisition on a laparoscopic suturing exercise after simulation training | Kilkenny et al. (64) | Vet surgery | 2019 | Canada | Simulator | Hybrid | Mixed | Dynamic | Undergraduate | None | Objective | N/A | Psychomotor skill | Surgery | Mixed |
| The effect of simulation training in anesthesia on student operational performance and patient safety | Jones et al. (65) | JVME | 2019 | USA | Simulator | High | VR | Dynamic | Undergraduate | Traditional | Objective | Better | Psychomotor skill | Clinical skills | Mixed |
| 3D anatomical model for teaching canine lumbosacral epidural anesthesia | Neves et al. (66) | ACTA CIRÚRGICA BRASILEIRA | 2020 | Brazil | Simulator | Low | 3D | Dynamic | Undergraduate | Traditional | Objective + questionnaire | Better | Psychomotor skill | Clinical skills | Dog |
| A patellar surgery haptic simulator for veterinary training | Lee et al. (67) | Journal of the Korea Computer Graphics Society | 2020 | Korea | Simulator | High | VR | Dynamic | Undergraduate | None | Objective + questionnaire | N/A | Psychomotor skill | Surgery | Dog |
| Comparison of a silicon skin pad and a tea towel as models for learning a simple interrupted suture | Baillie et al. (68) | JVME | 2020 | UK | Model | Low | Syntetic simple | Static | Undergraduate | Model | Objective + questionnaire | Same | Psychomotor skill | Surgery | Mixed |
| The effect of an ovariohysterectomy model practice on surgical times for final-year veterinary students’ first live-animal ovariohysterectomies | Annandale et al. (69) | JVME | 2020 | South Africa | Model | Low | Syntetic simple | Static | Undergraduate | Traditional | Questionnarie | Better | Psychomotor skill | Surgery | Dog |
| Development and validation of a canine castration model and rubric | Hunt et al. (70) | JVME | 2020 | USA | Model | Low | Syntetic simple | Static | Undergraduate | Traditional (live) | Objective + questionnaire | Better | Psychomotor skill | Surgery | Dog |
| Evaluation of a human virtual-reality endoscopy trainer for teaching early endoscopy skills to veterinarians | McCool et al. (71) | JVME | 2020 | USA | Simulator | High | VR | Dynamic | postgraduate | Traditional (live) | Objective + questionnaire | Same | Psychomotor skill | Clinical skills | Dog |
| Development and validation of a feline medial saphenous venipuncture model and rubric | Hunt et al. (72) | JVME | 2020 | USA | Simulator | Low | Syntetic simple | Dynamic | Under + post | None | Questionnarie | N/A | Psychomotor skill | Clinical skills | Cat |
| Evaluation of a laparoscopic abdominal simulator assessment to test readiness for laparoscopic ovariectomy in live dogs | French et al. (73) | Veterinary Surgery. | 2021 | US | Simulator | High | Hybrid | Dynamic | Undergraduate | None | Objective + questionnarie | Better | Psychomotor skills | Surgery | Dog |
| Case-based intensive veterinary radiology clerkship improves students' radiographic interpretation skills and overall course satisfaction scores | Gallastegui et al. (74) | Veterinary radiology | 2021 | US | Simulator | High | VR | Dynamic | Undergraduate | Traditional | Objective + questionnarie | Better | Knowledge | DI | Mixed |
| Evaluation of the Impact of Using a Simulator for Teaching Veterinary Students Cerebrospinal Fluid Collection: A Mixed-Methods Study | Langebæk et al. (75) | JVME | 2021 | Denmark | Simulator | High | Synthetic 3d | Dynamic | Undergraduate | None | Questionnarie | Same | Psychomotor skills | Clinical skills | Dog |
| Development and validation of a bovine castration model and rubric | Anderson et al. (76) | JVME | 2021 | USA | Model | Low | Syntetic simple | Static | Undergraduate | Traditional | Objective + questionnaire | Better | Psychomotor skill | Surgery | Bovine |
| Development of and Validity Evidence for a Canine Ocular Model for Training Novice Veterinary Students to Perform a Fundic Examination | Banse et al. (77) | JVME | 2021 | USA | Model | Low | Synthetic 3d | Static | Undergraduate | Traditional (live) | Objective | Same | Psychomotor skill | Clinical skills | Dog |
| Comparison of three canine models for teaching veterinary dental cleaning | Hunt et al. (78) | JVME | 2021 | USA | Model | Low | Synthetic 3d | Static | Undergraduate | Model | Questionnarie | Same/better | Psychomotor skill | Clinical skills | Dog |
| The influence of low-fidelity simulator training on canine peripheral venous puncture procedure | Da Silva et al. (79) | Veterinary world | 2021 | Brazil | Simulator | Low | Syntetic simple | Dynamic | Undergraduate | Traditional | Objective | Better | Psychomotor skill | Clinical skills | Dog |
| A simulated tumor for teaching principles of surgical oncology for biopsy and excision of skin and subcutaneous masses to veterinary students | Grimes et al. (80) | JVME | 2021 | USA | Model | Low | Syntetic simple | Dynamic | Undergraduate | None | Questionnarie | N/A | Psychomotor skill | Clinical skills | Dog |
| Effect of a spay simulator on student competence and anxiety | McArthur et al. (81) | JVME | 2021 | USA | Model | Low | Syntetic simple | Dynamic | Undergraduate | Traditional | Questionnarie | Better | Psychomotor skill | Surgery | Dog |
| Exploration of Immersive Virtual Reality in Teaching Veterinary Orthopedics | McCaw et al. (82) | JVME | 2022 | USA | Simulator | High | VR | Dynamic | Undergraduate | None | Questionnarie | Same | Knowledge | Anatomy/DI | Dog |
| Development and validation of a composed canine simulator for advanced veterinary laparoscopic training | Oviedo-Peñada et al. (83) | Frontiers | 2022 | Spain | Simulator | High | Synthetic simple | Dynamic | Postgraduate | None | Questionnarie | N/A | Psychomotor skills | Surgery | dog |
| Three-dimensional printed models of the heart represent an opportunity for inclusive learning | Borgeat et al. (84) | JVME | 2022 | UK | Model | Low | 3D printed | Static | Undergraduate | Traditional | Objective + questionnaire | Better | Knowledge | Cardiac anatomy / echocardiography | Dog |
| Enhancing Anatomical Instruction: Impact of a Virtual Canine Anatomy Program on Student Outcomes | Linton et al. (85) | Anatomical science education | 2022 | USA | Simulator | High | VR | Static | Undergraduate | Traditional | Objective + questionnaire | Same/better | Knowledge | Anatomy | Dog |
| Survey of students’ learning experience using a virtual slaughterhouse simulator in three UK veterinary schools during the COVID-19 pandemic | Garcia-Ara et al. (86) | Veterinary record | 2023 | UK | Simulator | High | VR | Dynamic | Undergraduate | None | Questionnarie | Same | Knowledge | Anatomy | Mixed |
| Use of 3D Printing Technology to Create a Canine Simulator for Cerebrospinal Fluid Sampling at the Lumbar Subarachnoid Space | Madden et al. (87) | JVME | 2023 | UK | Simulator | High | Synthetic 3d | Dynamic | Undergraduate | Traditional | Objective + questionnarie | Same | Knowledge+ physco | Clinical skills | Dog |
| Evaluation of a 3D computer model of the equine paranasal sinuses as a tool for veterinary anatomy | Canright et al. (88) | JVME | 2023 | UK | Model | Low | Synthetic simple | Static | Undergraduate | Traditional | Objective + questionnaire | Same | Knowledge | Anatomy | Horse |
| Implementation of a Canine Ergonomic Abdominal Simulator for Training Basic Laparoscopic Skills in Veterinarians | Hincapié-Gutiérrez et al. (89) | Animals (MDPI) | 2023 | Colombia | Simulator | Hybrid | Mixed | Dynamic | Under / Veterinarians | Traditional / other training | Objective + questionnaire | Same | Psychomotor skill | Surgery | Dog |
| Proficiency and Retention of Five Clinical Veterinary Skills Using Multipurpose Reusable Canine Manikins versus Live Animals: Model Development and Validation | Anderson et al. (90) | JVME | 2023 | USA | Simulator | Low | Synthetic simple (manikin) | Static | Undergraduate | Live animals | Objective + questionnaire | Same | Psychomotor skill | Clinical skills (5 skills) | Dog |
| Application of augmented reality models of canine skull in veterinary anatomical education | Jiang et al. (91) | anatomical science education | 2023 | China | Simulator | High | VR | Static | Undergraduate | Traditional | Objective + questionnaire | SAME | Knowledge | Anatomy | Dog |
| Significant transfer of surgical skills acquired in an advanced laparoscopic training program for total laparoscopic gastropexy assessed in a live porcine model | Oviedo-Peñata et al. (92) | Research square | 2023 | Spain | Simulator | High | Mixed | Dynamic | Postgraduate | Traditional | Objective + questionnaire | Better | Psychomotor skill | Surgery | Mixed |
| Interactive Virtual Reality with Educational Feedback Loops to Train and Assess Veterinary Students on the Use of Anesthetic Machine | Keets et al. (93) | JVME | 2024 | USA | Simulator | High | VR | Dynamic | Undergraduate | Traditional | Questionnarie | Same | Psychomotor skills | Anaesthesia | Mixed |
| SimuVet: a preliminary study of the innovative development of a simulator for epidural anesthesia training in dogs | Lobo Moraes et al. (94) | Frontiers | 2024 | Brazil | Simulator | High | Synthetic 3d | Dynamic | Postgraduate | None | Questionnarie | N/A | Psychomotor skills | Clinical skills | Dog |
| Development of a virtual reality simulator for training canine endotracheal intubation technique and evaluation of the educational impacts | Yamauchi et al. (95) | Vet J | 2024 | Japan | Simulator | High | VR | Dynamic | Undergraduate | Traditional video | Objective + questionnaire | Better | Psychomotor skill | Anaesthesia | Dog |
| Optimizing Student Outcomes: A Comparison of Two Teaching Methods for Identifying Vegetal Foreign Bodies in Canine Limbs Using Simulation Models and Ultrasound | Williams et al. (96) | Veterinary Radiology & Ultrasound | 2025 | Australia | Simulator | Low | Syntetic simple | Dynamic | Undergraduate | Model | objective + questionnarie | Same | Psychomotor skill | DI | Dog |
| Choose Your Own Adventure: Using Twine for Gamified Interactive Learning in Veterinary Anaesthesia | Redondo et al. (97) | Veterinary science | 2025 | Spain | Simulator | High | VR | Dynamic | Undergraduate | None | Questionnarie | Better | Knowledge | Anaesthesia | Dog and cat |
| Use of 3D printed low-cost models for veterinary clinical skills training | Hadžiomerović et al. (98) | Open Vet J | 2025 | Bosnia | Model | Low | Synthetic 3D | Static | Undergraduate | None | Questionnarie | Same | Knowledge | Anatomy | Dog |
| Anatomy of meat cuts: integrating 3D scanning and virtual reality in veterinary education and training | Hadžiomerović et al. (99) | Frontiers | 2025 | Turkiye | Simulator | Hybrid | 3D, synthetic, VR | Dynamic | Undergraduate | 3D, book, VR | Objective + questionnarie | 3D > trad > VR but VR medium term | Knowledge | clinical skills | Cow |
| Enhancing student performance with multicolored 3D printed neuroanatomical models in veterinary education | Hadžiomerović et al. (100) | BMC medical education | 2025 | Bosnia and Herzegovina | Model | Low | 3D | static | Undergraduate | Traditional | Objective + questionnaire | Better | Knowledge | Anatomy | Horse |
| Landmark Positioning on a Map: An Alternative Measure of Spatial Ability for Identifying Students Who May Benefit from Learning Gross Anatomy with Virtual Reality | Martin et al.  (101) | JVME | 2025 | USA | Simulator | High | VR | static | Undergraduate | 3D vs VR | Objective + questionnaire | Better/worse | Knowledge | Anatomy | Mixed |

Supplementary Table 1: classifies all the educational tools (includes original articles) integrating this review by types.

| **Article title** | **Authors** | **Year of publication** | **Country of first author’s affiliation** |
| --- | --- | --- | --- |
| Current concepts in simulation and other alternatives for veterinary education: a review | Valliyate et al. (109) | 2012 | India |
| The Use of Simulators for Teaching Practical Clinical Skills to Veterinary Students – A Review | Braid et al. (110) | 2022 | UK |
| If you build it, they will learn: A review of models in veterinary surgical education | Hunt et al. (111) | 2022 | USA |
| Links between learning goals, learning activities, and learning outcomes in simulation-based clinical skills training: a systematic review of the veterinary literature | Veenema et al. (112) | 2024 | Netherlands |
| The effect of simulator fidelity on procedure skill training: a literature review | Lefor et al. (113) | 2020 | Japan |
| Simulation models: another approach to teaching and learning | Casimir et al. (114) | 2023 | USA |
| Clinical skills of veterinary students – a cross-sectional study of the self-concept and exposure to skills training in Hannover, Germany | Rösch et al. (115) | 2014 | Germany |
| Veterinary anatomy teaching from real to virtual reality: An unprecedented shift during COVID-19 in socially distant era | Kapoor et al. (116) | 2022 | India |
| Surgeons’ Skills Training. In: *Small Animal Laparoscopy and Thoracoscopy* | Fransson et al. (117) | 2015 | USA |
| Validation of Training and Acquisition of Surgical Skills in Veterinary Laparoscopic Surgery: A Review | Oviedo-Peñata et al. (118) | 2020 | Colombia |
| Approaches to Laparoscopic Training in Veterinary Medicine: A Review of Personalized Simulators | Dejescu et al. (119) | 2023 | Romania |
| Take away body parts! An investigation into the use of 3D-printed anatomical models in undergraduate anatomy education | Smith et al. (120) | 2018 | UK |
| State of the Art and Future Prospects of Virtual and Augmented Reality in Veterinary Medicine: A Systematic Review | Aghapour et al. (121) | 2022 | Austria |
| Effective Use of Simulations for the Teaching and Acquisition of Veterinary Professional and Clinical Skills | Scalese et al. (122) | 2005 | USA |
| Integration of Virtual Patients in Education of Veterinary Medicine | Majernik et al. (123) | 2017 | Slovakia |
| Mixed Reality Applications in Tertiary Veterinary Education: A Systematic Review | Xu et al. (124) | 2023 | Ireland |
| Comparative Effectiveness of Training with Simulators Versus Traditional Instruction in Veterinary Education: Meta-Analysis and Systematic Review | Noyes et al. (125) | 2022 | USA |
| Introducing AI-generated cases (AI-cases) & standardized clients (AI-SCs) in communication training for veterinary students: perceptions and adoption challenges | Artemiou et al. (126) | 2025 | USA |
| Artificial intelligence—a valuable complementary tool for teaching and learning veterinary anatomy | Gadariya et al. | 2025 | India |

Supplementary Table 2: Published reviews of models and simulators used in veterinary education, included in figures 3 and 4 alongside the data from Supplementary Table 1.

REFERENCES:

1. Smeak DD, Beck ML, Shaffer CA, Gregg CG. Evaluation of Video Tape and a Simulator for Instruction of Basic Surgical Skills. *Veterinary Surgery* (1991) 20:30–36. doi: 10.1111/j.1532-950X.1991.tb00302.x

2. Smeak DD, Hill LN, Beck ML, Shaffer CA, Birchard SJ. Evaluation of an Autotutorial‐Simulator Program for Instruction of Hollow Organ Closure. *Veterinary Surgery* (1994) 23:519–528. doi: 10.1111/j.1532-950X.1994.tb00513.x

3. Olsen D, Bauer MS, Seim HB, Salman MD. Evaluation of a Hemostasis Model for Teaching Basic Surgical Skills. *Veterinary Surgery* (1996) 25:49–58. doi: 10.1111/j.1532-950X.1996.tb01376.x

4. Griffon DJ, Cronin P, Kirby B, Cottrell DF. Evaluation of a Hemostasis Model for Teaching Ovariohysterectomy in Veterinary Surgery. *Veterinary Surgery* (2000) 29:309–316. doi: 10.1053/jvet.2000.7541

5. Baillie S, Crossan A, Brewster S, Reid S. Preliminary Development and Evaluation of a Bovine Rectal Palpation Simulator for Training Veterinary Students. (2003)

6. Baillie S, Mellor DJ, Brewster SA, Reid SWJ. Integrating a Bovine Rectal Palpation Simulator into an Undergraduate Veterinary Curriculum. *Journal of Veterinary Medical Education* (2005) 32: doi: 10.3138/jvme.32.1.79

7. MORI T, ASANO K, KADOSAWA T, MOTIZUKI M, NISHIMURA R, MARUO K. Evaluation of a Dog Abdominal Surrogate Model for Teaching Basic Surgical Skills by Veterinary Students. *Journal of the Japan Veterinary Medical Association* (2006) 59:122–125. doi: 10.12935/jvma1951.59.122

8. Smeak DD. Teaching Surgery to the Veterinary Novice: The Ohio State University Experience. *Journal of Veterinary Medical Education* (2007) 34:620–627. doi: 10.3138/jvme.34.5.620

9. Bossaert P, Leterme L, Caluwaerts T, Cools S, Hostens M, Kolkman I, De Kruif A. Teaching Transrectal Palpation of the Internal Genital Organs in Cattle. *Journal of Veterinary Medical Education* (2009) 36:451–460. doi: 10.3138/jvme.36.4.451

10. Kinnison T, Forrest ND, Frean SP, Baillie S. Teaching bovine abdominal anatomy: use of a haptic simulator. *Anat Sci Educ* (2009) 2:280–285. doi: 10.1002/ase.109

11. Fransson BA, Ragle CA. Assessment of laparoscopic skills before and after simulation training with a canine abdominal model. *javma* (2010) 236:1079–1084. doi: 10.2460/javma.236.10.1079

12. Perez-Rivero JJ, Rendón-Franco E. Validation of the educational potential of a simulator to develop abilities and skills for the creation and maintenance of an intravenous cannula. *Altern Lab Anim* (2011) 39:257–260. doi: 10.1177/026119291103900310

13. Fletcher DJ, Militello R, Schoeffler GL, Rogers CL. Development and evaluation of a high-fidelity canine patient simulator for veterinary clinical training. *J Vet Med Educ* (2012) 39:7–12. doi: 10.3138/jvme.0711.073R

14. Lumbis RH, Gregory SP, Baillie S. Evaluation of a Dental Model for Training Veterinary Students. *Journal of Veterinary Medical Education* (2012) 39:128–135. doi: 10.3138/jvme.1011.108R

15. Lee S, Lee J, Lee A, Park N, Lee S, Song S, Seo A, Lee H, Kim J-I, Eom K. Augmented reality intravenous injection simulator based 3D medical imaging for veterinary medicine. *The Veterinary Journal* (2013) 196:197–202. doi: 10.1016/j.tvjl.2012.09.015

16. Gunning P, Smith A, Fox V, Bolt DM, Lowe J, Sinclair C, Witte TH, Weller R. Development and validation of an equine nerve block simulator to supplement practical skills training in undergraduate veterinary students. *Vet Rec* (2013) 172:450. doi: 10.1136/vr.101335

17. Eichel J-C, Korb W, Schlenker A, Bausch G, Brehm W, Delling U. Evaluation of a training model to teach veterinary students a technique for injecting the jugular vein in horses. *J Vet Med Educ* (2013) 40:288–295. doi: 10.3138/jvme.1012-09R1

18. Preece D, Williams SB, Lam R, Weller R. “Let’s get physical”: advantages of a physical model over 3D computer models and textbooks in learning imaging anatomy. *Anat Sci Educ* (2013) 6:216–224. doi: 10.1002/ase.1345

19. Fox V, Sinclair C, Bolt DM, Lowe J, Weller R. Design and Validation of a Simulator for Equine Joint Injections. *Journal of Veterinary Medical Education* (2013) 40:152–157. doi: 10.3138/jvme.0912-083R1

20. Usón-Gargallo J, Tapia-Araya AE, Díaz-Güemes Martin-Portugués I, Sánchez-Margallo FM. Development and Evaluation of a Canine Laparoscopic Simulator for Veterinary Clinical Training. *Journal of Veterinary Medical Education* (2014) 41:218–224. doi: 10.3138/jvme.0913-136R1

21. Usón-Gargallo J, Usón-Casaús JM, Pérez-Merino EM, Soria-Gálvez F, Morcillo E, Enciso S, Sánchez-Margallo FM. Validation of a Realistic Simulator for Veterinary Gastrointestinal Endoscopy Training. *Journal of Veterinary Medical Education* (2014) 41:209–217. doi: 10.3138/jvme.0913-127R

22. Williamson JA. Construct Validation of a Small-Animal Thoracocentesis Simulator. *Journal of Veterinary Medical Education* (2014) 41:384–389. doi: 10.3138/jvme.0314-037R

23. Williamson JA, Rito RMF. Development of a Training Model for Small Animal Thoracocentesis and Chest Tube Thoracostomy. *Altern Lab Anim* (2014) 42:201–205. doi: 10.1177/026119291404200307

24. Capilé KV, Campos GMB, Stedile R, Oliveira ST. Canine Prostate Palpation Simulator as a Teaching Tool in Veterinary Education. *J Vet Med Educ* (2015) 42:146–150. doi: 10.3138/jvme.1214-120R1

25. Giusto G, Comino F, Gandini M. Validation of an Effective, Easy-to-Make Hemostasis Simulator. *Journal of Veterinary Medical Education* (2015) 42:85–88. doi: 10.3138/jvme.0514-050R2

26. Williamson JA, Hecker K, Yvorchuk K, Artemiou E, French H, Fuentealba C. Development and validation of a feline abdominal palpation model and scoring rubric. *Veterinary Record* (2015) 177:151–151. doi: 10.1136/vr.103212

27. Nagel C, Ille N, Erber R, Aurich C, Aurich J. Stress Response of Veterinary Students to Gynaecological Examination of Horse Mares - Effects of Simulator-Based and Animal-Based Training. *Reprod Domest Anim* (2015) 50:866–871. doi: 10.1111/rda.12600

28. Nibblett BMD, Pereira MM, Williamson JA, Sithole F. Validation of a Model for Teaching Canine Fundoscopy. *Journal of Veterinary Medical Education* (2015) 42:133–139. doi: 10.3138/jvme.1014.100R

29. Troy JR, Bergh MS. Development and Efficacy of a Canine Pelvic Limb Model Used to Teach the Cranial Drawer and Tibial Compression Tests in the Stifle Joint. *Journal of Veterinary Medical Education* (2015) 42:127–132. doi: 10.3138/jvme.0914-094R1

30. Aulmann M, März M, Burgener IA, Alef M, Otto S, Mülling CKW. Development and Evaluation of Two Canine Low-Fidelity Simulation Models. *Journal of Veterinary Medical Education* (2015) 42:151–160. doi: 10.3138/jvme.1114-114R

31. Tapia-Araya AE, Usón-Gargallo J, Enciso S, Pérez-Duarte FJ, Díaz-Güemes Martin-Portugués I, Fresno-Bermejo L, Sánchez-Margallo FM. Assessment of Laparoscopic Skills in Veterinarians Using a Canine Laparoscopic Simulator. *Journal of Veterinary Medical Education* (2016) 43:71–79. doi: 10.3138/jvme.0315-034R1

32. Caston SS, Schleining JA, Danielson JA, Kersh KD, Reinertson EL. Efficacy of Teaching the Gambee Suture Pattern Using Simulated Small Intestine versus Cadaveric Small Intestine. *Veterinary Surgery* (2016) 45:1019–1024. doi: 10.1111/vsu.12554

33. Langebæk R, Toft N, Eriksen T. The SimSpay-Student Perceptions of a Low-Cost Build-It-Yourself Model for Novice Training of Surgical Skills in Canine Ovariohysterectomy. *J Vet Med Educ* (2015) 42:166–171. doi: 10.3138/jvme.1014-105

34. Read EK, Vallevand A, Farrell RM. Evaluation of Veterinary Student Surgical Skills Preparation for Ovariohysterectomy Using Simulators: A Pilot Study. *Journal of Veterinary Medical Education* (2016) 43:190–213. doi: 10.3138/jvme.0815-138R1

35. Fahie M, Cloke A, Lagman M, Levi O, Schmidt P. Training Veterinary Students to Perform Ovariectomy Using theMOOSE Spay Model with Traditional Method versus the Dowling Spay Retractor. *Journal of Veterinary Medical Education* (2016) 43:176–183. doi: 10.3138/jvme.0915-150R

36. Badman M, Tullberg M, Höglund OV, Hagman R. Veterinary Student Confidence after Practicing with a New Surgical Training Model for Feline Ovariohysterectomy. *J Vet Med Educ* (2016) 43:427–433. doi: 10.3138/jvme.1015-165R2

37. Levi O, Michelotti K, Schmidt P, Lagman M, Fahie M, Griffon D. Comparison between Training Models to Teach Veterinary Medical Students Basic Laparoscopic Surgery Skills. *Journal of Veterinary Medical Education* (2016) 43:80–87. doi: 10.3138/jvme.0715-109R

38. Williams DL, Wager C, Brearley J. Student attitudes regarding the educational value and welfare implications in the use of model eyes and live dogs in teaching practical fundus examination: evaluation of responses from 40 students. *Open Vet J* (2016) 6:172. doi: 10.4314/ovj.v6i3.5

39. Hage MCFNS, Massaferro AB, Lopes ÉR, Beraldo CM, Daniel J. Value of artisanal simulators to train veterinary students in performing invasive ultrasound-guided procedures. *Adv Physiol Educ* (2016) 40:98–103. doi: 10.1152/advan.00121.2015

40. Zolhavarieh S, Sadeghi-nasab A, Ghanbari S, Mirshokraei P, Ruhi athar M. Preliminary evaluation of learning performance of the simplest bovine trans-rectal palpation phantom for training veterinary students. *IRANIAN JOURNAL OF RUMINANTS HEALTH RESEARCH* (2016) 1:21–30.

41. Williamson JA, Dascanio JJ, Christmann U, Johnson JW, Rohleder B, Titus L. Development and Validation of a Model for Training Equine Phlebotomy and Intramuscular Injection Skills. *Journal of Veterinary Medical Education* (2016) 43:235–242. doi: 10.3138/jvme.0915-159R

42. Sidhu DS, Ruth JD, Lambert G, Rossmeisl JH. An easy to produce and economical three‐dimensional brain phantom for stereotactic computed tomographic‐guided brain biopsy training in the dog*. *Veterinary Surgery* (2017) 46:621–630. doi: 10.1111/vsu.12657

43. Allavena RE, Schaffer-White AB, Long H, Alawneh JI. Technical Skills Training for Veterinary Students: A Comparison of Simulators and Video for Teaching Standardized Cardiac Dissection. *Journal of Veterinary Medical Education* (2017) 44:620–631. doi: 10.3138/jvme.0516-095R

44. Nibblett BMD, Pereira MM, Sithole F, Orchard PAD, Bauman EB. Design and Validation of a Three-Dimensional Printed Flexible Canine Otoscopy Teaching Model. *Sim Healthcare* (2017) 12:91–95. doi: 10.1097/SIH.0000000000000227

45. Mariano Beraldo C, Rondon Lopes É, Hage R, Hage MCFNS. The value of homemade phantoms for training veterinary students in the ultrasonographic detection of radiolucent foreign bodies. *Advances in Physiology Education* (2017) 41:94–98. doi: 10.1152/advan.00163.2015

46. Little WB, Artemiou E, Conan A, Sparks C. Computer Assisted Learning: Assessment of the Veterinary Virtual Anatomy Education Software IVALA^TM^. *Veterinary Sciences* (2018) 5:58. doi: 10.3390/vetsci5020058

47. Christ R, Guevar J, Poyade M, Rea PM. Proof of concept of a workflow methodology for the creation of basic canine head anatomy veterinary education tool using augmented reality. *PLoS One* (2018) 13:e0195866. doi: 10.1371/journal.pone.0195866

48. Elarbi MM, Ragle CA, Fransson BA, Farnsworth KD. Face, construct, and concurrent validity of a simulation model for laparoscopic ovariectomy in standing horses. *Journal of the American Veterinary Medical Association* (2018) 253:92–100. doi: 10.2460/javma.253.1.92

49. Pérez-Merino EM, Usón-Gargallo J, Sánchez-Margallo FM, Usón-Casaús JM. Comparison of the use of fresh-frozen canine cadavers and a realistic composite ex vivo simulator for training in small animal flexible gastrointestinal endoscopy. *Journal of the American Veterinary Medical Association* (2018) 252:839–845. doi: 10.2460/javma.252.7.839

50. Li F, Liu C, Song X, Huan Y, Gao S, Jiang Z. Production of accurate skeletal models of domestic animals using three-dimensional scanning and printing technology. *Anat Sci Educ* (2018) 11:73–80. doi: 10.1002/ase.1725

51. Au Yong JA, Kim SE, Case JB. Survey of clinician and student impressions of a synthetic canine model for gastrointestinal surgery training. *Veterinary Surgery* (2019) 48:343–351. doi: 10.1111/vsu.13144

52. Mehrdadi S, Marjani M. Introduction and assessment of an inanimate model for basic surgical skills training of veterinary students. *J Surg Simul* (2018) 5:47–59. doi: 10.1102/2051-7726.2018.0006

53. Motta T, Carter B, Sweazy E, Taylor, McLoughlin M, Hill L. Development and validation of a low-fidelity orchiectomy simulator. *Clinical Theriogenology* (2018) 10:125–139.

54. Phillips H, Ellison GW, Mathews KG, Aronson LR, Schmiedt CW, Robello G, Selmic LE, Gregory CR. Validation of a model of feline ureteral obstruction as a tool for teaching microsurgery to veterinary surgeons. *Veterinary Surgery* (2018) 47:357–366. doi: 10.1111/vsu.12769

55. Jones JL, Rinehart J, Spiegel JJ, Englar RE, Sidaway BK, Rowles J. Teaching Tip: Development of Veterinary Anesthesia Simulations for Pre-Clinical Training: Design, Implementation, and Evaluation Based on Student Perspectives. *J Vet Med Educ* (2018) 45:232–240. doi: 10.3138/jvme.1016-163r

56. French HM, Dascanio JD, Peterson EW, Gilbert GE, Wright CC, Wickman DD, Bauman EB. Development and Student Evaluation of an Anatomically Correct High-Fidelity Calf Leg Model. *Journal of Veterinary Medical Education* (2018) 45:126–130. doi: 10.3138/jvme.0916-143r1

57. Annandale A, Annandale CH, Fosgate GT, Holm DE. Training Method and Other Factors Affecting Student Accuracy in Bovine Pregnancy Diagnosis. *Journal of Veterinary Medical Education* (2018) 45:224–231. doi: 10.3138/jvme.1016-166r1

58. Grimes JA, Wallace ML, Schmiedt CW, Parks AH. Evaluation of surgical models for training veterinary students to perform enterotomies. *Vet Surg* (2019) 48:985–996. doi: 10.1111/vsu.13228

59. Suñol A, Aige V, Morales C, López-Beltran M, Feliu-Pascual AL, Puig J. Use of Three-Dimensional Printing Models for Veterinary Medical Education: Impact on Learning How to Identify Canine Vertebral Fractures. *J Vet Med Educ* (2019) 46:523–532. doi: 10.3138/jvme.0817-109r

60. Xiberta P, Boada I. IVET, an Interactive Veterinary Education Tool. *Journal of Animal Science* (2019) 97:932–944. doi: 10.1093/jas/sky471

61. Chen C-Y, Elarbi M, Ragle CA, Fransson BA. Development and evaluation of a high-fidelity canine laparoscopic ovariectomy model for surgical simulation training and testing. *Journal of the American Veterinary Medical Association* (2019) 254:113–123. doi: 10.2460/javma.254.1.113

62. Au Yong JA, Case JB, Kim SE, Verpaalen VD, McConkey MJ. Survey of instructor and student impressions of a high‐fidelity model in canine ovariohysterectomy surgical training. *Veterinary Surgery* (2019) 48:975–984. doi: 10.1111/vsu.13218

63. Williamson JA, Brisson BA, Anderson SL, Farrell RM, Spangler D. Comparison of 2 canine celiotomy closure models for training novice veterinary students. *Veterinary Surgery* (2019) 48:966–974. doi: 10.1111/vsu.13224

64. Kilkenny JJ, White K, Singh A. Evaluating veterinary student skill acquisition on a laparoscopic suturing exercise after simulation training. *Vet Surg* (2019) 48:O66–O73. doi: 10.1111/vsu.12930

65. Jones JL, Rinehart J, Englar RE. The Effect of Simulation Training in Anesthesia on Student Operational Performance and Patient Safety. *Journal of Veterinary Medical Education* (2019) 46:205–213. doi: 10.3138/jvme.0717-097r

66. Neves EC das, Pelizzari C, Oliveira RS de, Kassab S, Lucas KDA, Carvalho YK de. 3D anatomical model for teaching canine lumbosacral epidural anesthesia. *Acta Cir Bras* (2020) 35:e202000608. doi: 10.1590/s0102-865020200060000008

67. Lee J, Eom K, Seo A. A patellar surgery haptic simulator for veterinary training. *Journal of the Korea Computer Graphics Society* (2020) 26:1–6.

68. Baillie S, Christopher R, Catterall AJ, Kruydenberg A, Lawrenson K, Wonham K, Kilfeather P, Warman S. Comparison of a Silicon Skin Pad and a Tea Towel as Models for Learning a Simple Interrupted Suture. *Journal of Veterinary Medical Education* (2020) 47:516–522. doi: 10.3138/jvme.2018-0001

69. Annandale A, Scheepers E, Fosgate GT. The Effect of an Ovariohysterectomy Model Practice on Surgical Times for Final-Year Veterinary Students’ First Live-Animal Ovariohysterectomies. *Journal of Veterinary Medical Education* (2020) 47:44–55. doi: 10.3138/jvme.1217-181r1

70. Hunt JA, Heydenburg M, Kelly CK, Anderson SL, Dascanio JJ. Development and Validation of a Canine Castration Model and Rubric. *Journal of Veterinary Medical Education* (2020) 47:78–90. doi: 10.3138/jvme.1117-158r1

71. McCool KE, Bissett SA, Hill TL, Degernes LA, Hawkins EC. Evaluation of a Human Virtual-Reality Endoscopy Trainer for Teaching Early Endoscopy Skills to Veterinarians. *Journal of Veterinary Medical Education* (2020) 47:106–116. doi: 10.3138/jvme.0418-037r

72. Hunt J, Hughes C, Asciutto M, Johnson J. Development and validation of a feline medial saphenous venipuncture model and rubric. *Journal of Veterinary Medical Education* (2020) 47:333–341.

73. French ED, Griffon DJ, Kass PH, Fahie MA, Gordon-Ross P, Levi O. Evaluation of a laparoscopic abdominal simulator assessment to test readiness for laparoscopic ovariectomy in live dogs. *Veterinary Surgery* (2021) 50:O49–O66. doi: 10.1111/vsu.13604

74. Gallastegui A, Spoldi E, Billhymer AC, Stefanou CR. Case-based intensive veterinary radiology clerkship improves students’ radiographic interpretation skills and overall course satisfaction scores. *Vet Radiol Ultrasound* (2022) 63:138–147. doi: 10.1111/vru.13043

75. Langebæk R, Berendt M, Tipold A, Engelskirchen S, Dilly M. Evaluation of the Impact of Using a Simulator for Teaching Veterinary Students Cerebrospinal Fluid Collection: A Mixed-Methods Study. *J Vet Med Educ* (2021) 48:217–227. doi: 10.3138/jvme.2019-0006

76. Anderson SL, Miller L, Gibbons P, Hunt JA, Roberson J, Raines JA, Patterson G, Dascanio JJ. Development and Validation of a Bovine Castration Model and Rubric. *Journal of Veterinary Medical Education* (2021) 48:96–104. doi: 10.3138/jvme.2018-0016

77. Banse HE, McMillan CJ, Warren AL, Hecker KG, Wilson B, Skorobohach BJ, Carter RT, Lewin AC, Kondro DA, Ungrin MD, et al. Development of and Validity Evidence for a Canine Ocular Model for Training Novice Veterinary Students to Perform a Fundic Examination. *Journal of Veterinary Medical Education* (2021) 48:620–628. doi: 10.3138/jvme-2020-0035

78. Hunt JA, Schmidt P, Perkins J, Newton G, Anderson SL. Comparison of Three Canine Models for Teaching Veterinary Dental Cleaning. *Journal of Veterinary Medical Education* (2021) 48:573–583. doi: 10.3138/jvme-2020-0001

79. Da Silva DAF, Fernandes AA, Ventrone AE, Dias A, Silveira AMS, Santarém CL, Ribeiro GGDS, Nogueira RMB. The influence of low-fidelity simulator training on canine peripheral venous puncture procedure. *Vet World* (2021) 14:410–418. doi: 10.14202/vetworld.2021.410-418

80. Grimes JA, Appleton KL, Moss LA, Bullington A-CM. A Simulated Tumor for Teaching Principles of Surgical Oncology for Biopsy and Excision of Skin and Subcutaneous Masses to Veterinary Students. *Journal of Veterinary Medical Education* (2021) 48:636–639. doi: 10.3138/jvme-2020-0028

81. MacArthur SL, Johnson MD, Colee JC. Effect of a Spay Simulator on Student Competence and Anxiety. *Journal of Veterinary Medical Education* (2021) 48:115–128. doi: 10.3138/jvme.0818-089r3

82. McCaw K, West A, Duncan C, Frey D, Duerr F. Exploration of Immersive Virtual Reality in Teaching Veterinary Orthopedics. *J Vet Med Educ* (2022) 49:716–720. doi: 10.3138/jvme-2021-0009

83. Oviedo-Peñata CA, Giraldo Mejía GE, Riaño-Benavides CH, Maldonado-Estrada JG, Lemos Duque JD. Development and validation of a composed canine simulator for advanced veterinary laparoscopic training. *Front Vet Sci* (2022) 9:936144. doi: 10.3389/fvets.2022.936144

84. Borgeat K, Shearn AIU, Payne JR, Hezzell M, Biglino G. Three-Dimensional Printed Models of the Heart Represent an Opportunity for Inclusive Learning. *J Vet Med Educ* (2022) 49:346–352. doi: 10.3138/jvme-2020-0141

85. Linton A, Garrett AC, Ivie KR, Jones JD, Martin JF, Delcambre JJ, Magee C. Enhancing Anatomical Instruction: Impact of a Virtual Canine Anatomy Program on Student Outcomes. *Anatomical Sciences Education* (2021) doi: 10.1002/ase.2087

86. Garcia-Ara A, Sandoval-Barron E, Seguino A. Survey of students’ learning experience using a virtual slaughterhouse simulator in three UK veterinary schools during the COVID-19 pandemic. *Veterinary Record* (2023) 193:e3307. doi: 10.1002/vetr.3307

87. Madden M, Collins R, Schwarz T, Suñol A. Use of 3D Printing Technology to Create a Canine Simulator for Cerebrospinal Fluid Sampling at the Lumbar Subarachnoid Space. *J Vet Med Educ* (2022)e20210159. doi: 10.3138/jvme-2021-0159

88. Canright A, Bescoby S, Dickson J. Evaluation of a 3D Computer Model of the Equine Paranasal Sinuses as a Tool for Veterinary Anatomy Education. *Journal of Veterinary Medical Education* (2023) 50:234–242. doi: 10.3138/jvme-2021-0134

89. Hincapié-Gutiérrez LC, Oviedo-Peñata CA, Rojas-Galvis MA, Riaño-Benavides CH, Maldonado-Estrada JG. Implementation of a Canine Ergonomic Abdominal Simulator for Training Basic Laparoscopic Skills in Veterinarians. *Animals* (2023) 13:1140. doi: 10.3390/ani13071140

90. Anderson LS, Olin SJ, Whittemore JC. Proficiency and Retention of Five Clinical Veterinary Skills Using Multipurpose Reusable Canine Manikins versus Live Animals: Model Development and Validation. *J Vet Med Educ* (2023) 50:654–660. doi: 10.3138/jvme-2022-0103

91. Jiang N, Jiang Z, Huang Y, Sun M, Sun X, Huan Y, Li F. Application of augmented reality models of canine skull in veterinary anatomical education. *Anat Sci Educ* (2024) 17:546–557. doi: 10.1002/ase.2372

92. Oviedo-Peñata CA, Lemus-Duque JD, Maldonado-Estrada JG. Significant transfer of surgical skills acquired in an advanced laparoscopic training program for total laparoscopic gastropexy assessed in a live porcine model. (2023) doi: 10.21203/rs.3.rs-2548173/v1

93. Keets L, Boscan P, Arakaki L, Schraeder B, Tornatzky C, Vans M, Jiang W, Rao S. Interactive Virtual Reality with Educational Feedback Loops to Train and Assess Veterinary Students on the Use of Anesthetic Machine. *Journal of Veterinary Medical Education* (2024) 51:412–420. doi: 10.3138/jvme-2022-0140

94. Lobo Moraes P, Ghisi L, Paes de Barros AJB, de Carvalho Peixoto VH, Brandini Népoli PE, Moleta Colodel E, Souza de Lima LF, Lopes de Souza R. SimuVet: a preliminary study of the innovative development of a simulator for epidural anesthesia training in dogs. *Front Vet Sci* (2024) 11:1322871. doi: 10.3389/fvets.2024.1322871

95. Yamauchi A, Oshita R, Kudo A, Umezawa M, Shimizu R, Kamo S, Fujita Y, Takagi S. Development of a virtual reality simulator for training canine endotracheal intubation technique and evaluation of the educational impacts. *The Veterinary Journal* (2024) 307:106203. doi: 10.1016/j.tvjl.2024.106203

96. Williams A, Schoenfeld E, Callcott E, Rotne R. Optimizing Student Outcomes: A Comparison of Two Teaching Methods for Identifying Vegetal Foreign Bodies in Canine Limbs Using Simulation Models and Ultrasound. *Veterinary Radiology & Ultrasound* (2025) 66:e70073. doi: 10.1111/vru.70073

97. Redondo JI, Marti-Scharfhausen MR, Martínez-Albiñana A, Cañón-Pérez A, Gutiérrez-Bautista ÁJ, Viscasillas J, Hernández-Magaña EZ. Choose Your Own Adventure: Using Twine for Gamified Interactive Learning in Veterinary Anaesthesia. *Veterinary Sciences* (2025) 12:156. doi: 10.3390/vetsci12020156

98. Hadžiomerović N, Šunje-Rizvan A, Maksimović A, Šatrović L, Tandir F. Use of 3D printed low-cost models for veterinary clinical skills training. *Open Vet J* (2025) 15:863–870. doi: 10.5455/OVJ.2025.v15.i2.35

99. Hadžiomerović N, Čaklovica K, Dučić N, Gjoni Gündemir M, Vejzović A, Fazlović N, Avdić R, Čaklovica F, Tandir F. Anatomy of meat cuts: integrating 3D scanning and virtual reality in veterinary education and training. *Front Vet Sci* (2025) 12:1680785. doi: 10.3389/fvets.2025.1680785

100. Hadžiomerović N, Avdić R, Muminović AJ, Muftić A, Pandžić A, Tandir F, Vejzović A, Gündemir O, Hadžiomerović AI. Enhancing student performance with multicolored 3D printed neuroanatomical models in veterinary education. *BMC Med Educ* (2025) 25:1323. doi: 10.1186/s12909-025-07908-y

101. Martin JF, Linton A, Svenson GR, Garrett AC, Mango DW, Svec PM, Magee C. Landmark Positioning on a Map: An Alternative Measure of Spatial Ability for Identifying Students Who May Benefit from Learning Gross Anatomy with Virtual Reality. *Journal of Veterinary Medical Education* (2025) 52:615–623. doi: 10.3138/jvme-2024-0011

102. Baillie S, Crossan A, Brewster SA, May SA, Mellor DJ. Evaluating an Automated Haptic Simulator Designed for Veterinary Students to Learn Bovine Rectal Palpation. *Simulation in Healthcare* (2010) 5:261. doi: 10.1097/SIH.0b013e3181e369bf

103. Clausse M, Nejamkin P, Bulant CA, Genaro A, Landivar F, Del Sole MJ, Clausse A. A low-cost portable simulator of a domestic cat larynx for teaching endotracheal intubation. *Veterinary Anaesthesia and Analgesia* (2020) 47:676–680. doi: 10.1016/j.vaa.2020.05.006

104. Chen C-Y, Ragle CA, Lencioni R, Fransson BA. Comparison of 2 training programs for basic laparoscopic skills and simulated surgery performance in veterinary students. *Veterinary Surgery* (2017)

105. Balsa IM, Giuffrida MA, Culp WTN, Mayhew PD. Perceptions and experience of veterinary surgery residents with minimally invasive surgery simulation training. *Veterinary Surgery* (2020) 49:O21–O27. doi: 10.1111/vsu.13295

106. Spruijt A, Prins-Aardema CC, Antonio de Carvalho-Filho M, Jaarsma D, Martin A. Co-constructive Veterinary Simulation: A Novel Approach to Enhancing Clinical Communication and Reflection Skills. *J Vet Med Educ* (2023) 50:134–139. doi: 10.3138/jvme-2021-0160

107. Fransson BA, Ragle CA, Bryan ME. Effects of two training curricula on basic laparoscopic skills and surgical performance among veterinarians. *Journal of the American Veterinary Medical Association* (2012) 241:451–460. doi: 10.2460/javma.241.4.451

108. Grevemeyer B, Knight A. The Development of a Clinical Skills Laboratory at Ross University School of Veterinary Medicine. *Altern Lab Anim* (2018) 46:177–183. doi: 10.1177/026119291804600305

109. Valliyate M, Robinson NG, Goodman JR. Current concepts in simulation and other alternatives for veterinary education: a review. *Veterinární medicína* (2012) 57:325–337. doi: 10.17221/6261-VETMED

110. Braid HR. The Use of Simulators for Teaching Practical Clinical Skills to Veterinary Students - A Review. *Altern Lab Anim* (2022) 50:184–194. doi: 10.1177/02611929221098138

111. Hunt JA, Simons MC, Anderson SL. If you build it, they will learn: A review of models in veterinary surgical education. *Vet Surg* (2022) 51:52–61. doi: 10.1111/vsu.13683

112. Veenema NJ, Hierck BP, Bok HGJ, Salvatori DCF. Links between learning goals, learning activities, and learning outcomes in simulation-based clinical skills training: a systematic review of the veterinary literature. *Front Vet Sci* (2024) 11:1463642. doi: 10.3389/fvets.2024.1463642

113. Lefor AK, Harada K, Kawahira H, Mitsuishi M. The effect of simulator fidelity on procedure skill training: a literature review. *Int J Med Educ* (2020) 11:97–106. doi: 10.5116/ijme.5ea6.ae73

114. Casimir R, Linn L, King H, McKenzie D, Thompson M, Perry RL. Simulation models: another approach to teaching and learning. *Journal of the American Veterinary Medical Association* (2023) 261:47–47. doi: 10.2460/javma.22.11.0509

115. Rösch T, Schaper E, Tipold A, Fischer MR, Dilly M, Ehlers JP. Clinical skills of veterinary students - a cross-sectional study of the self-concept and exposure to skills training in Hannover, Germany. *BMC Vet Res* (2014) 10:969. doi: 10.1186/s12917-014-0302-8

116. Kapoor K, Singh A. Veterinary anatomy teaching from real to virtual reality: An unprecedented shift during COVID-19 in socially distant era. *Anat Histol Embryol* (2022) 51:163–169. doi: 10.1111/ahe.12783

117. Fransson BA, Towle Millard HA, Ragle CA. “Surgeons’ Skills Training,.” *Small Animal Laparoscopy and Thoracoscopy*. (2015). p. 1–11 doi: 10.1002/9781118845912.ch1

118. Oviedo-Peñata CA, Tapia-Araya AE, Lemos JD, Riaño-Benavides C, Case JB, Maldonado-Estrada JG. Validation of Training and Acquisition of Surgical Skills in Veterinary Laparoscopic Surgery: A Review. *Front Vet Sci* (2020) 7:306. doi: 10.3389/fvets.2020.00306

119. Dejescu CA, Bel LV, Melega I, Muresan SMC, Oana LI. Approaches to Laparoscopic Training in Veterinary Medicine: A Review of Personalized Simulators. *Animals* (2023) 13:3781. doi: 10.3390/ani13243781

120. Smith CF, Tollemache N, Covill D, Johnston M. Take away body parts! An investigation into the use of 3D-printed anatomical models in undergraduate anatomy education. *Anat Sci Educ* (2018) 11:44–53. doi: 10.1002/ase.1718

121. Aghapour M, Bockstahler B. State of the Art and Future Prospects of Virtual and Augmented Reality in Veterinary Medicine: A Systematic Review. *Animals (Basel)* (2022) 12:3517. doi: 10.3390/ani12243517

122. Scalese RJ, Issenberg SB. Effective Use of Simulations for the Teaching and Acquisition of Veterinary Professional and Clinical Skills. *Journal of Veterinary Medical Education* (2005) 32:461–467. doi: 10.3138/jvme.32.4.461

123. Majernik J, Maďar M, Mojžišová J. Integration of Virtual Patients in Education of Veterinary Medicine. *Annals of Computer Science and Information Systems*. (2017). p. 185–188 https://annals-csis.org/Volume_11/drp/134.html [Accessed September 3, 2025]

124. Xu X, Kilroy D, Kumar A, Iqbal MZ, Mangina E, Campbell AG. “Mixed Reality Applications in Tertiary Veterinary Education: A Systematic Review.,” In: Cai Y, Mangina E, Goei SL, editors. *Mixed Reality for Education*. Singapore: Springer Nature (2023). p. 241–264 doi: 10.1007/978-981-99-4958-8_10

125. Noyes JA, Carbonneau KJ, Matthew SM. Comparative Effectiveness of Training with Simulators Versus Traditional Instruction in Veterinary Education: Meta-Analysis and Systematic Review. *J Vet Med Educ* (2022) 49:25–38. doi: 10.3138/jvme-2020-0026

126. Artemiou E, Hooper S, Dascanio L, Schmidt M, Gilbert G. Introducing AI-generated cases (AI-cases) & standardized clients (AI-SCs) in communication training for veterinary students: perceptions and adoption challenges. *Front Vet Sci* (2025) 11: doi: 10.3389/fvets.2024.1504598
